# DKK3-LRP1 complex and a chemical inhibitor regulate Aβ clearance in models of Alzheimer’s disease

**DOI:** 10.1126/sciadv.adz2099

**Published:** 2025-11-07

**Authors:** Ruihan Yang, Lin Wang, Yue Li, Jian Zhu, Juxian Wang, David Schlessinger, Jian Sima

**Affiliations:** ^1^ Laboratory of Aging Neuroscience and Neuropharmacology, School of Basic Medicine and Clinical Pharmacy, China Pharmaceutical University, Nanjing 210009, China.; ^2^ Department of Psychology, Eastern Illinois University, Charleston, IL 61920, USA.; ^3^ Institute of Medicinal Biotechnology, Chinese Academy of Medical Science and Peking Union Medical College, Beijing 100050, China.; ^4^ Laboratory of Genetics and Genomics, NIA/NIH-IRP, 251 Bayview Blvd, Room 10B014, Baltimore, MD 21224, USA.

## Abstract

Impaired clearance of amyloid-β (Aβ) contributes to Alzheimer’s disease (AD) pathogenesis, but its upstream modulators remain poorly defined. We report secreted Dickkopf (DKK) proteins—DKK1 through DKK4—as previously unrecognized ligands of low-density lipoprotein receptor–related protein 1 (LRP1), a principal Aβ clearance receptor. Analyses of cells derived from a patient with AD, postmortem tissue, and 5×FAD mice reveal that DKK1 and DKK3 are elevated in AD and reduce Aβ uptake and degradation in neurons and astrocytes. Mechanistically, DKKs inhibit Aβ clearance by competitively binding LRP1 and promoting its internalization. In 5×FAD mice, DKK3 overexpression worsens, while knockout improves, Aβ pathology and cognitive outcomes. A targeted high-throughput screen of ~3000 compounds identified SJ-300 as a potent and selective inhibitor of the DKK3-LRP1 interaction. SJ-300 restores Aβ clearance and rescues cognitive function and neuropathology in 5×FAD mice. These findings uncover DKK3-LRP1 axis as a contributor for Aβ metabolism and nominate SJ-300 as a promising therapeutic candidate for AD intervention.

## Teaser

Targeting DKK3-LRP1 interaction restores Aβ clearance and improves cognitive function in Alzheimer’s disease mouse models.

## INTRODUCTION

Accumulation, aggregation, and deposition of amyloid-β (Aβ) peptides in the brain have long been recognized as associated with Alzheimer’s disease (AD) pathogenesis. Recent evidence suggests that augmented Aβ levels primarily result from impaired clearance, rather than overproduction, in both common sporadic and late-onset forms of AD (*1*). Given that Aβ deposition can precede clinical symptoms in asymptomatic individuals by many years (*2*), targeting the Aβ clearance pathway presents a promising strategy to reduce Aβ accumulation and potentially delay or even prevent disease progression. In recent years, several monoclonal antibodies (mAbs) targeting Aβ have been approved for AD treatment, with additional antibodies showing potential for approval. These mAbs consistently reduce brain Aβ aggregates by more than 70%, as detected by positron emission tomography (PET) (*3*, *4*). Notably, mAb-mediated Aβ clearance involves multiple pathways, including microglial phagocytosis, neuronal endocytosis, and potentially astrocytic uptake (*4*), further highlighting the Aβ clearance pathway as a viable therapeutic target. However, alternative approaches, such as small-molecule compounds that target Aβ clearance, remain underexplored.

Aβ clearance occurs through two primary mechanisms: proteolytic degradation and receptor-mediated clearance (*5*). Targeting Aβ-degrading enzymes has demonstrated efficacy in attenuating Aβ pathology and cognitive deficits in AD animal models (*6*). However, receptor-mediated clearance, which involves complex interactions between neurons, glial cells, the blood-brain barrier (BBB), interstitial fluid flow, perivascular drainage, and the lymphatic system, remains less well understood (*5*, *7*). Low-density lipoprotein receptor–related protein 1 (LRP1) is considered a key receptor in Aβ endocytosis and subsequent cellular degradation (*8*, *9*). LRP1 also facilitates the transcytosis of Aβ across the BBB, with subsequent clearance via peripheral organs such as the liver, spleen, and kidney (*10*, *11*). LRP1 is ubiquitously expressed in brain cells and binds a variety of ligands, including Aβ, amyloid precursor protein (APP), apolipoprotein E, β_2_-macroglobulin, and receptor-associated protein (RAP), all of which function in Aβ production or clearance (*12*). While the potential ligands and their impact on LRP1-mediated Aβ metabolism remain largely unknown, targeting LRP1 or its regulatory pathways may offer therapeutic possibility for AD. However, because of its multifunctionality and widespread expression in various organs, concerns about drug specificity and potential side effects have been raised.

DKK1, one of the Dickkopf family proteins (DKKs) and comprising four homologous members (DKK1, 2, 3, and 4), has also been implicated in AD progression (*13*, *14*). The DKKs are known to regulate the Wnt signaling pathway, which plays a crucial role in cellular processes such as fate determination, organ development, and tissue homeostasis (*15*). DKK1, 2, and 4, but not DKK3, are identified to competitively inhibit the interaction of Wnt ligands with LRP5/6 receptors (*16*–*18*), key components of the low density lipoprotein (LDL) receptor family, thus blocking the activation of the Wnt/β-catenin signaling pathway. Elevated expression of DKK1, as well as dysregulation of Wnt signaling, has been associated with the pathogenesis of AD, contributing not only to Aβ deposition and neuronal apoptosis but also to disrupted neurogenesis and synaptic plasticity (*14*, *19*). This highlights the importance of DKK-LRP interactions in both maintaining physiological homeostasis and contributing to disease progression.

We present here a role for secreted DKKs in AD by acting as LRP1 ligands that inhibit Aβ clearance. In the 5×FAD mouse model, DKK3 knockout (KO)—the only DKK member not directly affecting Wnt signaling—reduces Aβ pathology and improves cognition. These findings highlight the DKK3-LRP1 interaction as a potential therapeutic target for AD. Supporting this, we identify SJ-300, a compound that blocks this interaction, enhancing Aβ clearance and alleviating AD-related cognitive and pathological deficits. Our results propose SJ-300 as a promising therapeutic candidate, targeting a mechanism distinct from traditional Wnt signaling.

## RESULTS

### DKKs directly bind to membrane LRP1

While DKKs are known to bind LRP5/6 and modulate Wnt signaling, recent studies have suggested that DKKs may have broader functions in other contexts, including tumor progression and AD (*20*). We hypothesized that DKKs might have Wnt-independent functions by interacting with other membrane (Mem) receptors. To investigate this, we developed a strategy to purify DKK4-containing protein complexes (fig. S1A) using a validated immunoprecipitation (IP)–based method (*21*).

We generated a stable human embryonic kidney (HEK) 293 cell line (“DKK4^+^”) expressing Flag-tagged DKK4. Total cell extracts from DKK4^+^ cells were fractionated using gel-filtration chromatography, and peak fractions containing DKK4 (peak 1 in fig. S1B) were subjected to IP using either an anti-Flag or anti-DKK4 antibody. Mass spectrometry (MS) analysis of the immunoprecipitated samples confirmed the presence of DKK4 along with its known receptors, LRP5/6. MS also detected a comparable abundance of LRP1, another member of the LRP family (fig. S1C). The elution profile of LRP1 in the gel-filtration assay indicated its involvement in protein complexes (fig. S1B), and subsequent co-IP confirmed that endogenous LRP1 directly associates with DKK4 (fig. S1D). Crucially, this interaction between DKK4 and LRP1 was independent of LRP5/6, as no secondary interaction between DKK4 and LRP5/6 was required for the formation of the DKK4-LRP1 complex. In addition, consistent with our previous reports on DKK4-LRP6 complex formation (*22*), only full-length (FL) DKK4 was capable of forming a complex with LRP1 (fig. S1D).

Using LRP1 minireceptor constructs (mLRP1-I to -IV), which selectively express each of the four ligand-binding repeat units of LRP1 (I, II, III, and IV) (*23*), we observed that DKK4 interacted with all four repeats (fig. S1E). In parallel assays with the other three members of the DKK family, DKK2 and DKK3 also strongly bound to repeats II, III, and IV. In contrast, DKK1 exhibited a more restricted binding profile, interacting predominantly with repeats II and IV (fig. S1F). These findings suggest that DKKs may engage in Wnt-independent interactions with LRP1, providing additional insights into their potential roles beyond traditional Wnt signaling.

### DKK1 and DKK3 form complexes with LRP1 in human and mouse brain

**Fig. 1.**
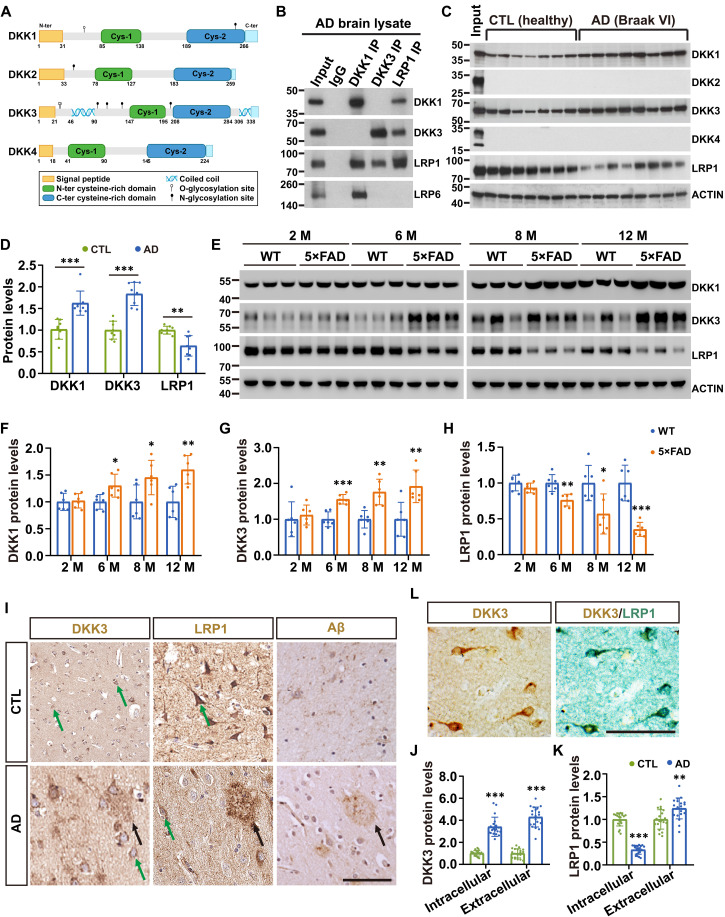
Interaction of DKK family proteins with LRP1 in AD brains. (**A**) Schematic representation of human DKK protein structures. N-ter, N-terminal; C-ter, C-terminal. (**B**) IP and subsequent immunoblotting analysis of AD brain cell lysates using antibodies specific to DKK1, DKK3, and LRP1. Controls include 5% of the total lysate as input and immunoglobulin G (IgG) as a negative isotype control. (**C**) Immunoblotting of brain lysates from seven healthy controls (CTL) and eight patients with AD (Braak stage VI) using antibodies against DKK1 to 4 and LRP1, with cell lysates from HEK293 cells transfected with DKK-expressing plasmids serving as input control. (**D**) Histogram depicting the average density of protein bands for CTL and AD samples from (C), with CTL protein levels normalized to 1.0. (**E**) Immunoblot analysis of LRP1, DKK3, and DKK1 in hippocampal lysates from wild-type (WT) and 5×FAD mice, aged 2 to 12 months. M, months. (**F** to **H**) Quantification of band intensities from (E) and fig. S2A (total of six mice; *n* = 6), with WT protein levels normalized to 1.0. (**I**) DAB immunohistochemistry (IHC) illustrating the expression patterns of DKK3, LRP1, and Aβ in adjacent cortical paraffin sections from CTL and patients with AD. Nuclei are counterstained with hematoxylin (blue). Green arrows indicate intracellular IHC signal; black arrows point to extracellular signal. Scale bar, 200 μm. DAB, 3,3′-diaminobenzidine. (**J** and **K**) Bar graphs showing the intracellular and extracellular expression levels of DKK3 (J) and LRP1 (K) from (I), with CTL normalized to 1.0. (**L**) Dual-color IHC staining for LRP1 (brown) and DKK3 (green) in cortical tissue from patients with AD. Scale bar, 50 μm. IHC assays analyzed *n* ≥ 20 slices per group. All data are presented as mean ± SD. Statistical significance was assessed using Student’s *t* test, with *P < 0.05, **P < 0.01, and ***P < 0.001.

Using human brain lysates, co-IP experiments confirmed the formation of DKK1-LRP1 and DKK3-LRP1 complexes. As previously reported (*24*), DKK3, with its distinct protein structure (Fig. 1A), did not bind to LRP6 (Fig. 1B), further supporting its unique interaction profile within the DKK family. Given the emerging role of LRP1 in brain Aβ metabolism and AD pathogenesis (*8*), we next examined the expression levels of DKK proteins in human brain tissue. Brain tissues from seven healthy control individuals and eight patients with AD, sourced from the Harvard Brain Collection, were lysed and immunoblotted with antibodies specific for LRP1 or each of the DKKs. Notably, DKK2 and DKK4 were undetectable in these brain samples, while both DKK1 and DKK3 were found to be expressed at higher levels in AD brains compared to controls (Fig. 1, C and D), consistent with previous reports (*19*, *25*). In contrast, LRP1 expression was reduced in AD samples (Fig. 1, C and D), in agreement with findings from a larger cohort study (*26*). These results suggest that the up-regulation of DKK1 and DKK3, along with the down-regulation of LRP1, may play a role in the altered molecular landscape of AD.

Next, we collected hippocampal tissues from 2-, 6-, 8-, and 12-month-old wild-type (WT) and 5×FAD mice, lysed the samples, and performed immunoblotting analyses. The data revealed that the levels of DKK1, DKK3, and LRP1 in the brains of 2-month-old WT and 5×FAD mice were similar. However, as the mice aged, we observed a progressive decrease in LRP1 levels in the AD mice, while both DKK1 and DKK3 levels were consistently elevated at all time points (Fig. 1, E to H, and fig. S2A).

We further investigated the localization of LRP1 and DKK3 in samples from patients with AD and 5×FAD mice. Immunohistochemical (IHC) analysis of adjacent cortical sections confirmed the presence of Aβ plaques exclusively in AD brains, as expected (Fig. 1I). In accord with the immunoblotting results, intracellular LRP1 expression (green arrows; Fig. 1I) was markedly reduced from patients with AD compared to healthy controls. However, in AD samples, LRP1 signal was also detected surrounding extracellular Aβ deposits (black arrows; Fig. 1I), suggesting a potential redistribution of LRP1 in association with amyloid pathology (Fig. 1, I and K). Immunofluorescence (IF) analysis in mouse hippocampus revealed that LRP1 is broadly expressed across multiple cell types, including neurons, astrocytes, and microglia (fig. S2, B to D). Notably, DKK3 expression was substantially elevated within cells in AD brain sections and, unlike in controls, was also localized around extracellular Aβ plaques (Fig. 1, I and J), supporting a pathological association between DKK3 and Aβ plaques. Dual-color IHC further demonstrated colocalization of LRP1 and DKK3 in cortical cells from patients with AD (Fig. 1L). Similarly, in 5×FAD mouse brains, IHC revealed decreased LRP1 expression accompanied by increased levels of both DKK1 and DKK3. As in human samples, LRP1 colocalized with either DKK1 or DKK3 in hippocampal cells (fig. S3). Together, our results support the association of DKK1 and DKK3 with LRP1 in AD pathology and suggest a potential role for these proteins in modulating LRP1-mediated processes.

### DKK1 and DKK3 bind to each of the ligand-binding repeats of LRP1 with distinct affinities

**Fig. 2.**
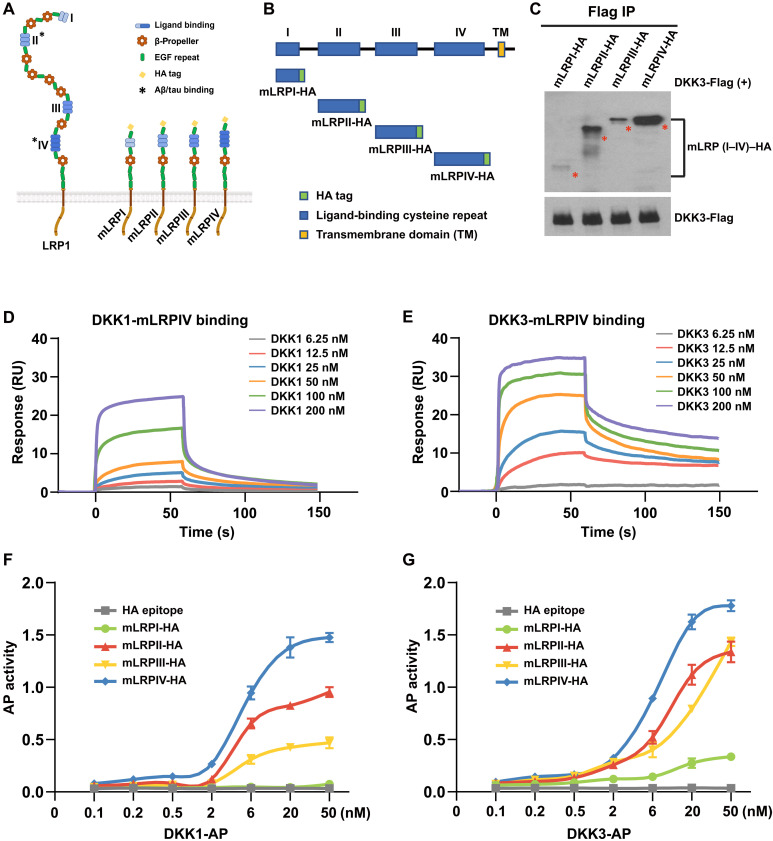
Differential binding affinity of DKK to ligand-binding repeat (I to IV) of LRP1. (**A**) Schematic representation of the full LRP1 structure and the minireceptor mLRPI to IV. Created in BioRender [R. Yang (2025); https://BioRender.com/do57arf]. (**B**) Detailed schematic depicting each of the ligand-binding repeats (I to IV) of LRP1, with each repeat protein synthesized with a C-terminal HA tag. (**C**) Immunoblot demonstrating the direct protein-protein interaction of purified DKK3 with each of the I to IV domains of LRP1 following Flag-tag IP. Experiments used 5 nM DKK3-Flag and each mLRPI to IV domain protein. (**D** and **E**) SPR analysis showing the binding dynamics of DKK1 and DKK3 to mLRPIV. The response curves represent varying concentrations of DKK1 and DKK3. (**F** and **G**) Assessment of AP binding affinity with quantification of AP enzymatic activity at 650-nm absorbance, detailing the interaction of dosed DKK1-AP or DKK3-AP with 5 nM mLRPI, II, III, or IV. RU, resonance unit.

DKK1, DKK2, and DKK4 interact directly with the extracellular epidermal growth factor (EGF)/YWTD repeats of LRP5/6 (*27*), moieties homologous to LRP1 (repeats I to IV). To determine whether DKK1 or DKK3 also bind to LRP1 (I to IV), we generated individual LRP1 (I to IV) peptides using minireceptors (shown in Fig. 2A) as polymerase chain reaction (PCR) templates and fused with a hemagglutinin (HA) tag at C terminus (Fig. 2B) in a cell-free protein synthesis system. DKK1 and DKK3, tagged with alkaline phosphatase (AP) and 6 × His from HEK293-conditioned medium (CM), as well as prokaryotically expressed mLRPIV–6 × His (a truncated form of LRP1 that mimics FL LRP1 function), were produced and purified using Ni–nitrilotriacetic acid (NTA) Agarose beads. Protein purity was confirmed via silver-stained gels and immunoblotting (fig. S4). In vitro protein-protein binding assays further confirmed direct interactions of DKK1 and DKK3 with each of the I to IV repeats of LRP1, with varying binding affinities, as shown by IP and immunoblotting (Fig. 2C). To quantitatively assess the binding affinities of DKK1 and DKK3 for mLRPIV, surface plasmon resonance (SPR) was used. Purified mLRPIV–6 × His was covalently immobilized on a CM5 sensor chip, and SPR analyses revealed strong binding of both DKK1 and DKK3 to mLRPIV, with dissociation constants (*K*_d_) of 82.3 and 35.5 nM, respectively. Notably, DKK3 exhibited a stronger affinity for mLRPIV compared to DKK1 (Fig. 2, D and E). Enzymatic AP activity assays further quantified the differential binding affinities of DKK1 and DKK3 to the LRP1 repeats. DKK1 preferentially bound to repeats II, III, and IV in the order IV > II > III, whereas DKK3 bound to repeats IV, II, and III in the same order, with additional binding to repeat I at a lower affinity (IV > II/III > I) (Fig. 2, F and G). These results demonstrate that DKK1 and DKK3 can bind to ligand-binding repeats of LRP1, with distinct binding profiles and affinities.

LRP1 has been identified as a membrane receptor for DKK3, the atypical member of the DKK family that does not interact with the Wnt receptor LRP5/6. We infer that LRP1 function as a membrane receptor for DKKs, particularly DKK1 and DKK3, may be intricately connected to the pathogenesis of AD.

### DKK1 and DKK3 each inhibit LRP1-mediated endocytosis and cellular clearance of Aβ

**Fig. 3.**
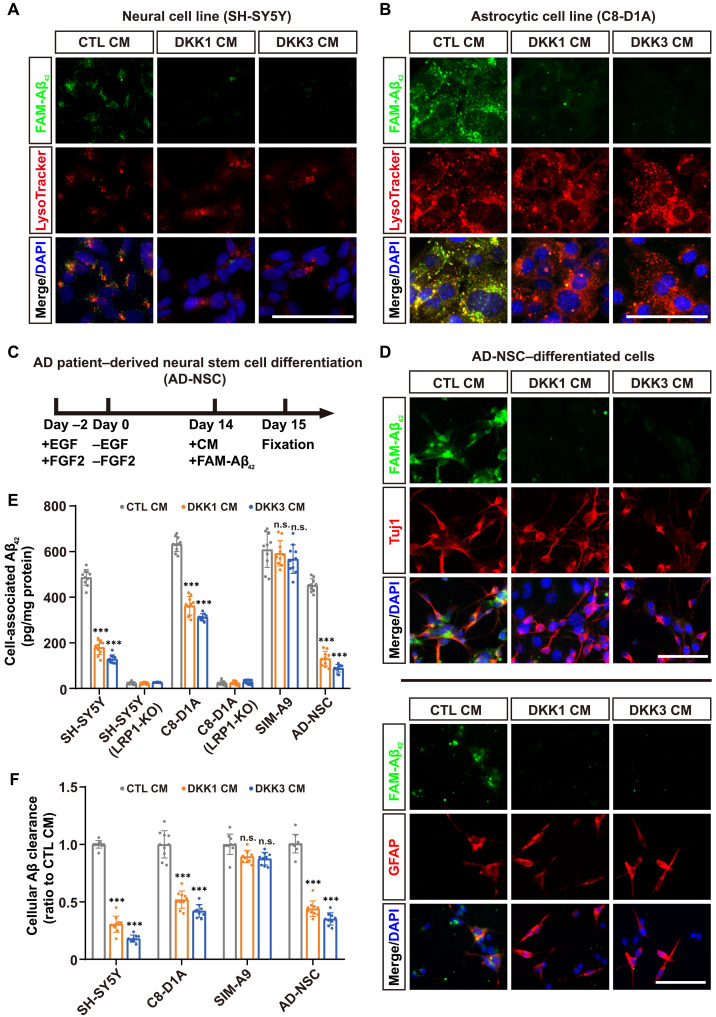
Inhibition of LRP1-mediated Aβ uptake and clearance by DKK1 and DKK3. (**A** and **B**) Subcellular colocalization of fluorescently labeled Aβ_42_ (FAM-Aβ_42_; green) with LysoTracker (red) in SH-SY5Y neuroblastoma cells and C8-D1A astrocytic cells following treatment with CM as indicated. Nuclei are counterstained with 4′,6-diamidino-2-phenylindole (DAPI; blue). Scale bars, 50 μm. (**C**) Schematic of the experimental protocol used for culturing and differentiating neural stem cells derived from a patient with AD (AD-NSCs). (**D**) Images showing internalization of FAM-Aβ_42_ (green) in differentiated AD-NSCs, colabeled with Tuj1 for neurons (red; top) or glial fibrillary acidic protein (GFAP) for astrocytes (red; bottom) under various CM treatments. Scale bar, 50 μm. Tuj1, β-III tubulin. (**E**) ELISA quantification of Aβ_42_ uptake in cells treated with CTL, DKK1, or DKK3 CM containing soluble Aβ_42_. (**F**) Similar setup as in (E) but with Aβ_42_ preincubated for 2 hours to allow for internalization, followed by a wash and an additional 8-hour incubation in fresh medium. ELISA quantified the decrease in internalized Aβ_42_ as an estimate of cellular Aβ clearance (see Materials and Methods for details). Data are presented as mean ± SD. Statistical significance determined by Student’s *t* test, with **P < 0.01, ***P < 0.001, and n.s. (not significant).

To determine the impact of DKK1 and DKK3 on LRP1-mediated Aβ clearance, we used a previously described cell-based Aβ uptake assay (*28*). We conducted initial evaluations of Aβ endocytosis in various brain-derived cell lines including SH-SY5Y (neuroblastoma), C8-D1A (astrocytes), and SIM-A9 (microglia). LRP1-deficient lines—PEA13 fibroblasts, SH-SY5Y (LRP1-KO), and C8-D1A (LRP1-KO)—were used as negative controls. Verification of cellular morphology, marker expression, and LRP1 immunoblotting confirmed the cell identity (fig. S5, A to C). Following this, cells were incubated with green fluorescent carboxyfluorescein (FAM)–Aβ_42_ for 24 hours, leading to successful internalization into lysosomal compartments in WT SH-SY5Y and C8-D1A cells, but was markedly absent in LRP1-deficient lines (fig. S5D), consistent with the established role of LRP1 in mediating Aβ endocytosis (*28*, *29*). To further explore the effects of DKK1 and DKK3 on Aβ uptake, cells were pretreated for 2 hours with CM devoid of DKKs (control CM), enriched with DKK1 (DKK1 CM) or DKK3 (DKK3 CM), followed by exposure to FAM-Aβ_42_ for an additional 24 hours. Notably, internalized FAM-Aβ_42_ levels were markedly reduced in neuronal and astrocytic cells treated with either DKK1 CM or DKK3 CM (Fig. 3, A and B). To rule out potential confounding effects from the CM itself, C8-D1A astrocytes were treated with recombinant DKK1 or DKK3 proteins (50 nM; based on Fig. 2), which produced a comparable reduction in Aβ uptake to that observed with DKK-enriched CM (fig. S5E). Enzyme-linked immunosorbent assay (ELISA) analysis confirmed that cell-associated Aβ_42_ was markedly decreased following treatment with DKK1 or DKK3 in these cell lines (Fig. 3E). Neither DKK1 nor DKK3 markedly inhibited Aβ_42_ endocytosis in microglial cells (Fig. 3E and fig. S5F), suggesting that Aβ uptake in microglia may involve more complex or redundant LRP1-related pathways (*30*–*32*) or alternative endocytic and phagocytic pathways independent of DKKs that remain to be fully characterized (*33*).

We further analyzed the effects of DKKs in brain cells derived from a patient with AD. Initially, induced pluripotent stem cell (iPSC)–derived neural stem cells (AD-NSCs) of a patient with AD were cultured and differentiated as described (Fig. 3C). IF staining with a nestin marker at day 0 confirmed the identity of the cells as NSCs. After 14 days of differentiation, more than 90% of the cells had matured into neurons and astrocytes, as determined by β-III tubulin (Tuj1) and glial fibrillary acidic protein (GFAP) staining (fig. S5G). Subsequent analyses using Aβ uptake and ELISA assays revealed that both DKK1 and DKK3 inhibited Aβ endocytosis in differentiated neurons and astrocytes. Notably, DKK3 exhibited a stronger inhibitory effect compared to DKK1 (Fig. 3, D and E).

To assess whether internalized Aβ was subsequently degraded, we used an established cellular Aβ clearance assay (*28*, *29*). Quantitative analysis of Aβ clearance revealed a reduction in cells cultured with DKK1 CM or DKK3 CM compared to those in control CM (Fig. 3F). Again, DKK3 exerted a stronger inhibitory effect than DKK1, consistent with its higher binding affinity to LRP1 (Fig. 2). Thus, these results suggest that both DKK1 and DKK3 can inhibit LRP1-mediated Aβ uptake and subsequent clearance in neuronal and astrocytic cells.

### DKK1 and DKK3 each inhibit Aβ binding to membrane LRP1

**Fig. 4.**
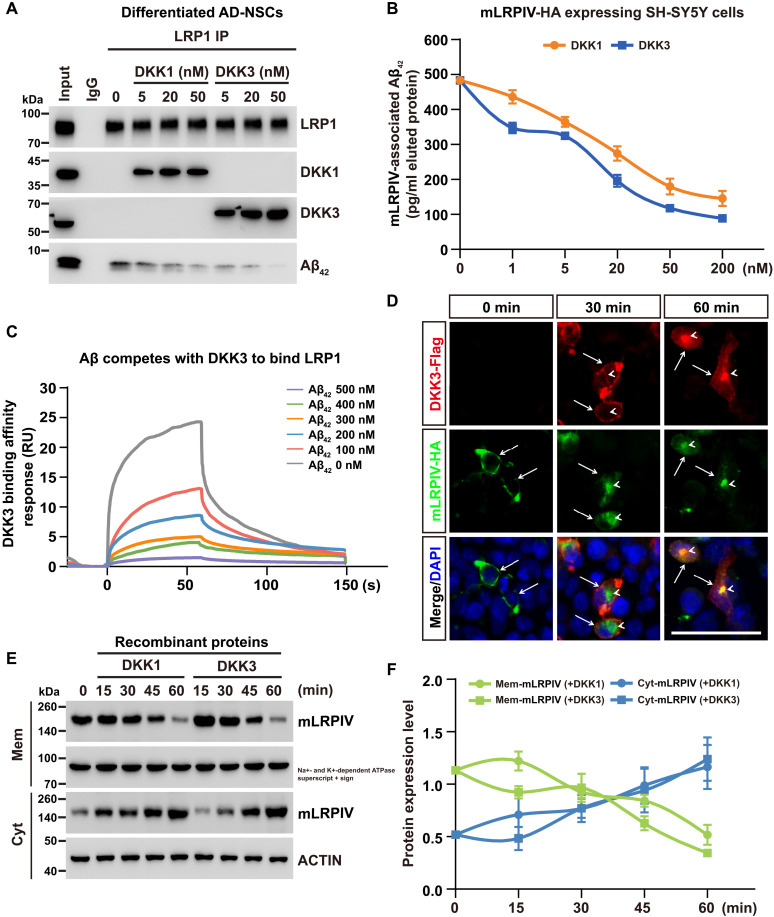
Dual mechanisms control DKK inhibition of LRP1-mediated Aβ uptake. (**A**) Differentiated AD-NSCs were incubated with 1 μM Aβ_42_ and various concentrations of purified DKK1-Flag or DKK3-Flag for 30 min, followed by IP of LRP1 and immunoblotting using specific antibodies. Five percent of the total lysate was used as input, with IgG serving as a negative CTL. (**B**) SH-SY5Y cells stably expressing mLRPIV treated as in (A) but with an expanded range of DKK1 or DKK3 concentrations. Levels of Aβ_42_ following HA-tag IP were quantified using ELISA. (**C**) SPR analysis depicting the competitive binding dynamics of Aβ_42_ to mLRPIV in the presence of 35.5 nM DKK3. The assay was conducted with varying concentrations of Aβ_42_. (**D**) IF imaging demonstrating the localization of DKK3-Flag (red) and mLRPIV-HA (green) in SH-SY5Y cells at 0, 30, and 60 min posttreatment with DKK3-Flag CM. Arrow indicates cell membrane localization; arrowhead points to intracellular localization. Nuclei are counterstained with DAPI (blue). Scale bar, 50 μm. (**E**) Immunoblot analysis showing levels of Mem and Cyt mLRPIV in SH-SY5Y cells after treatment with 50 nM recombinant DKK1 or DKK3 at specified time points. Na^+^- and K^+^-dependent ATPase (Na^+^,K^+^-ATPase) and ACTIN were used as loading controls for membrane and cytoplasmic proteins, respectively. (**F**) Quantification of band density for proteins shown in (E). Membrane and cytoplasmic proteins were normalized to Na^+^,K^+^-ATPase or ACTIN, respectively. Error bars represent mean ± SD from biological triplicates.

Previous studies have indicated that soluble Aβ can directly or indirectly bind to LRP1 on the cell surface, facilitating its endocytosis and subsequent metabolic degradation (*34*). Our results extend these findings by demonstrating that DKK1 and DKK3 can also bind strongly to the II and IV repeats of LRP1 (Fig. 2). To evaluate whether DKKs affect Aβ binding to LRP1, we treated differentiated AD-NSC cultures with 1 μM Aβ_42_ along with varying concentrations of purified DKK1 or DKK3 for an additional 30 min. Total protein extracts were then immunoprecipitated using an anti-LRP1 antibody. Immunoblot analysis revealed that treatment with DKK1 or DKK3 did not alter the levels of endogenous LRP1 protein; however, the association of Aβ_42_ with LRP1 decreased in a DKK dose-dependent manner (Fig. 4A).

To further quantify the effects of DKKs, we engineered a recombinant SH-SY5Y cell line to stably express mLRPIV-HA, a functional equivalent of LRP1. These cells were incubated with Aβ_42_ and varying concentrations of DKKs, ranging from 1 to 200 nM. After treatment, total proteins were extracted, and mLRPIV-HA was immunoprecipitated using an anti-HA affinity gel. The eluted proteins were subjected to Aβ_42_ ELISA, revealing that mLRPIV-associated Aβ_42_ levels decreased progressively with higher doses of DKK1 or DKK3. Notably, treatment with 20 nM of DKK1 resulted in ~44% reduction in Aβ_42_ binding to mLRPIV, while the same concentration of DKK3 achieved a reduction of about 60% (Fig. 4B). Complementary SPR experiments confirmed that Aβ can compete with DKK3 to bind LRP1 (Fig. 4C), in line with the direct interaction of Aβ and LRP1 (*35*, *36*). These results corroborate the hypothesis that DKK3 effectively blocks the association of Aβ with LRP1.

### DKK1 and DKK3 can promote LRP1 internalization independent of Aβ

DKK1 can promote the internalization of its receptor LRP6 (*37*, *38*). We hypothesized that DKKs might similarly induce the internalization of LRP1 from the cell membrane into the cytoplasm (Cyt). To investigate this, we transfected SH-SY5Y cells with mLRPIV-HA expressing plasmids and treated these cells with DKK3-Flag CM. IF imaging initially showed mLRPIV at the cell membrane in untreated cells (0 min) (Fig. 4D, left). However, following a 30-min treatment with DKK3, we observed a decrease in membrane-associated mLRPIV and an increase in its cytoplasmic localization, with notable colocalization with DKK3 (Fig. 4D, middle). By 60 min, most mLRPIV and DKK3 were colocalized in large punctate structures within the cytoplasm (Fig. 4D, right). To further validate these observations, we cultured additional mLRPIV-expressing SH-SY5Y cells, treated them with 50 nM recombinant DKK1 or DKK3 proteins, and then performed subcellular fractionation to separate Mem and Cyt proteins. Immunoblot analysis confirmed that treatment with DKK1 or DKK3 led to a time-dependent decrease in Mem-mLRPIV and an increase in Cyt-mLRPIV (Fig. 4, E and F). These results conclusively demonstrate that DKKs can promote LRP1 internalization from the membrane into the cytoplasm, independent of Aβ presence.

Overall, our findings support a dual mechanism for DKK-mediated inhibition of LRP1-mediated Aβ uptake. DKKs not only compete with Aβ for binding to membrane-bound LRP1 but also promote the internalization of LRP1. These combined actions effectively reduce Aβ interaction with its receptor on the cell surface.

### DKK3 KO alleviates cognitive deficits and Aβ pathologies in 5×FAD mice

**Fig. 5.**
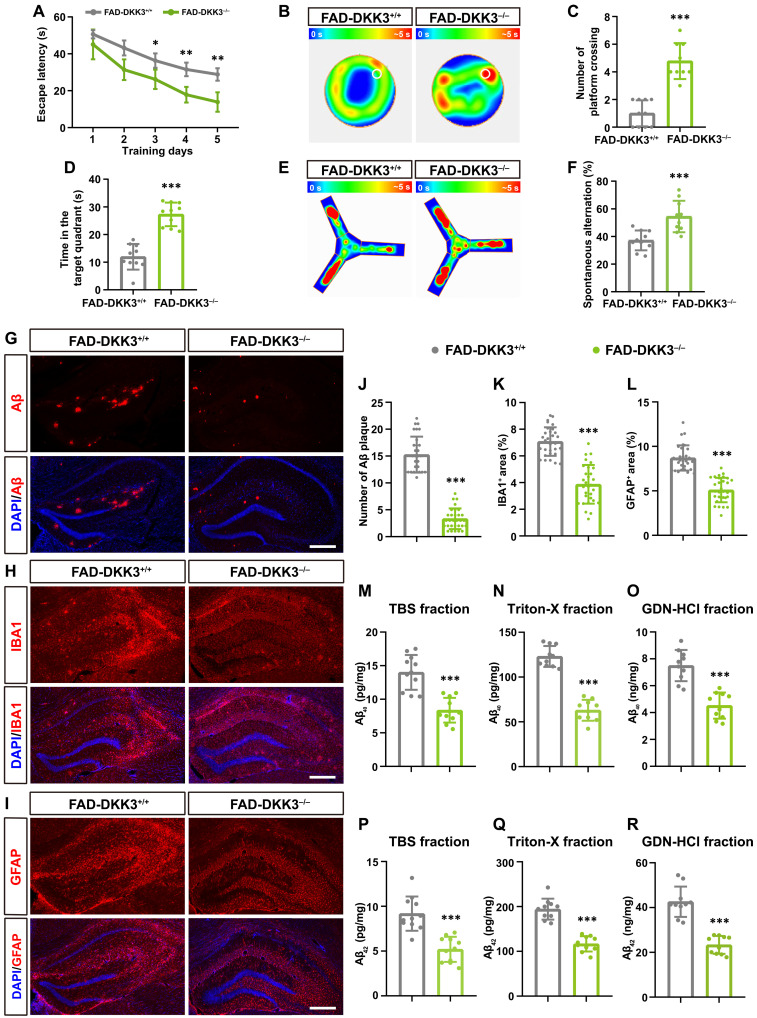
DKK3 KO alleviates cognitive deficits and Aβ pathologies in 5×FAD mice. (**A**) Escape latency of FAD-DKK3^+/+^ (control) and FAD-DKK3^−/−^ (KO) mice over 5 days of MWM training. (**B**) Representative heatmap images from the probe trials of the MWM on day 6, with the platform location indicated as a white circle. (**C**) Bar graph quantifying the number of platform crossings. (**D**) Bar graph showing the time spent in the quadrant containing the platform. (**E**) Heatmaps illustrating the frequency of visits in the Y-maze test. (**F**) Bar graph quantifying the percentage of spontaneous alternation in the Y-maze. (**G** to **I**) IHC staining of hippocampal sections for Aβ (red), GFAP (red), and IBA1 (red) in FAD-DKK3^+/+^ and FAD-DKK3^−/−^ mice, with nuclei counterstained using DAPI (blue). Scale bars, 500 μm. IBA1, ionized calcium-binding adapter molecule 1. (**J** to **L**) Bar graphs showing quantification of the total number of Aβ plaques (J), the percentage area positive for IBA1 (K), and the percentage area positive for GFAP (L). (**M** to **O**) ELISA measurements of Aβ_40_ levels in tris-buffered saline (TBS), Triton-X, and guanidine (GDN) fractions extracted from the brains of 6-month-old FAD-DKK3^+^/^+^ and FAD-DKK3−/− mice. (**P** to **R**) Similar to (M) to (O), detailing the levels of Aβ_42_ in the corresponding fractions. Behavioral tests and Aβ ELISA assays included *n* = 10 mice per group; IHC assays analyzed *n* ≥ 30 slices randomly selected from at least 6 mice per group. Data are presented as mean ± SD. Statistical significance was evaluated using Student’s *t* test, with *P < 0.05, **P < 0.01, and ***P < 0.001.

The DKK3-KO mice are viable and fertile, with no major alterations observed in organ development and physiology (*39*), supporting a Wnt-independent function of DKK3. To explore the impact of DKK3 ablation on AD pathology, we crossed 5×FAD mice with DKK3^+/−^ mice to generate 5×FAD mice on a DKK3^−/−^ genetic background (FAD-DKK3^−/−^). Cognitive functions were assessed using the Morris water maze (MWM) and Y-maze tests. In the MWM, FAD-DKK3^−/−^ mice exhibited enhanced cognitive abilities compared to FAD-DKK3^+/+^ (control) mice, demonstrating reduced latency during the acquisition phase (Fig. 5A) and increased time spent in the target quadrant along with more platform crossings during the probe trials (Fig. 5, B to D). Similarly, Y-maze tests indicated improved spatial working memory in FAD-DKK3^−/−^ mice, as evidenced by a higher spontaneous alternation rate compared to control FAD mice (Fig. 5, E and F). In addition, to determine whether DKK3 KO affects cognitive function in mice with WT background, we compared WT with DKK3^−/−^ mice in the absence of the 5×FAD transgene. Results from the MWM and Y-maze showed no obvious differences between the two groups (fig. S7), suggesting that DKK3 plays a regulatory role specifically in the context of AD pathology.

IHC analyses were performed to assess Aβ plaque deposition, microglial clustering, and astrogliosis in the hippocampi of 6-month-old mice. Our data indicated that DKK3 KO substantially reduced the number of Aβ plaques by 78.1%, the area of ionized calcium-binding adapter molecule 1 (IBA1^+^) microglia (microgliosis) by 45.2%, and the area of GFAP^+^ astrocytes (astrogliosis) by 41.4% (Fig. 5, G to L). To further evaluate the levels of soluble and insoluble Aβ, brain tissues were lysed using a sequential extraction method based on differential solubility in tris-buffered saline (TBS), TBS plus 1% Triton X-100 (Triton-X), and guanidine hydrochloride (GDN-HCl) (*40*). ELISA results revealed that DKK3 KO led to a reduction in the levels of both Aβ_40_ and Aβ_42_ across all three fractions (Fig. 5, M to R). Thus, our findings conclusively demonstrate that DKK3 KO alleviates both the cognitive and pathological deficits observed in 5×FAD mice.

### DKK3 overexpression exacerbates cognitive decline and pathological deficits in 5×FAD mice

**Fig. 6.**
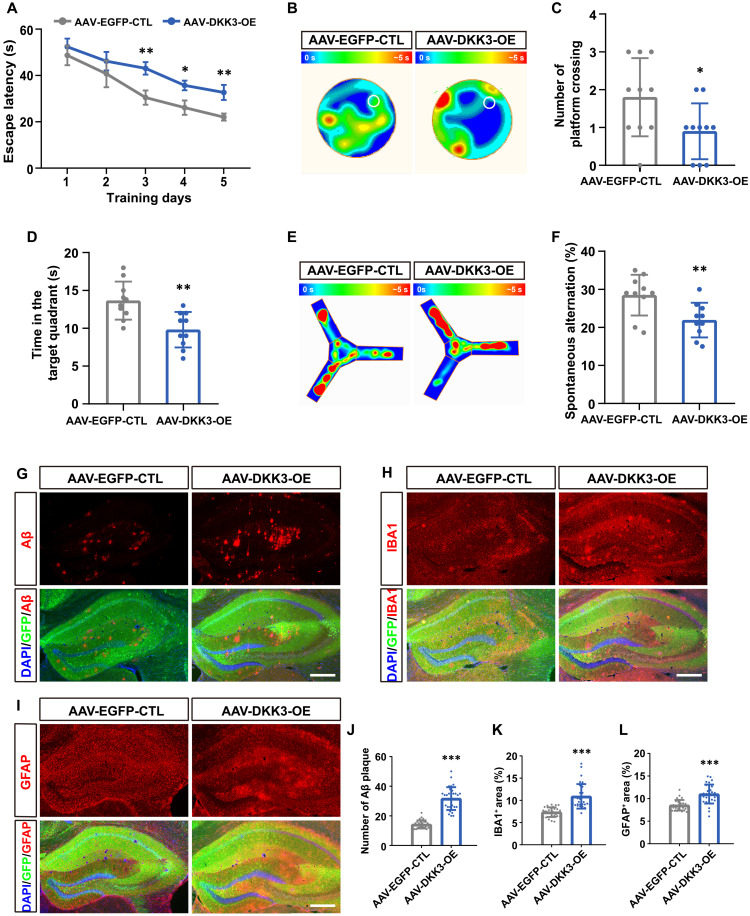
DKK3-OE aggravates cognitive and pathological deficits in 5×FAD mice. (**A**) Escape latency of 5×FAD mice over 5 days of training in groups with AAV-mediated DKK3-OE (AAV-DKK3-OE) versus control (AAV-EGFP-CTL). (**B**) Heatmaps of mouse trajectories during probe trials on day 6 of the MWM tests, with the platform location indicated as a white circle. Quantitation showing the number of crossing the platform (**C**) and time spent (**D**) in the platform located quadrant. (**E**) Heatmaps indicating the frequency of visits by mice in the Y-maze test. (**F**) Quantification of the percentage of spontaneous alternation in Y-maze. (**G** to **I**) IHC staining (red) of Aβ, GFAP, and IBA1 in hippocampi of AAV-DKK3-OE and AAV-EGFP-CTL mice, with nuclei counterstained using DAPI (blue). Scale bars, 500 μm. Quantification showing the total number of Aβ plaques (**J**), the percentage area positive for IBA1 (**K**), and the percentage area positive for GFAP (**L**). Behavioral tests included *n* = 10 mice per group; IHC assays analyzed *n* ≥ 30 slices randomly selected from at least 6 mice per group. Data are presented as mean ± SD. Statistical significance was evaluated using Student’s *t* test, with *P < 0.05, **P < 0.01, and ***P < 0.001.

To confirm the detrimental role of DKK3 in AD, we used an adeno-associated virus (AAV) tool, previously established in our group (*41*), to overexpress DKK3 (DKK3-OE) in the hippocampi of 5×FAD mice. The schema of AAV construction and experimental procedure is detailed in fig. S6 (A and B). Green fluorescent protein (GFP) imaging of brain coronal sections demonstrated extensive viral transduction across the hippocampal region (fig. S6C). 8 weeks (W) post- AAV injection, we assessed the cognitive function of the 5×FAD mice. In contrast to the improvements observed in DKK3-KO mice, 5×FAD mice with DKK3-OE displayed impaired spatial learning and memory in both the MWM and Y-maze tests compared to control AAV-treated mice (Fig. 6, A to F). Notably, similar to DKK3 KO, DKK3-OE in WT mice had no observable impact on cognitive performance (fig. S7), further supporting that the detrimental effects of DKK3 are specific to the AD pathological context. In conjunction with the behavioral assessments, we evaluated the impact of DKK3-OE on Aβ-associated pathological features. Our analyses revealed that DKK3-OE substantially exacerbated AD pathology: There was a 123.0% increase in the number of Aβ plaques (Aβ^+^), a 48.4% increase in microgliosis (IBA1^+^ area), and a 28.9% increase in astrogliosis (GFAP^+^ area) (Fig. 6, G to L). These results underscore that DKK3-OE not only impairs cognitive function but also intensifies AD pathology in 5×FAD mice, implicating the DKK3 and possibly the DKK3-LRP1 complex as critical contributors to AD pathogenesis.

### Identifying compounds that inhibit DKK-LRP1 complexes

To counteract the inhibitory effect of DKKs on Aβ clearance, we initiated a screening for small chemical compounds that could disrupt the DKK-LRP1 interaction. We used an AP cell surface–binding assay (*22*) to quantify the interactions between DKK-LRP1 complexes. HEK293 cells transfected with either an empty vector or vectors expressing LRP6 or the minireceptor forms of LRP1 (mLRPI to mLRPIV) were incubated with CM containing either DKK1-AP or DKK3-AP. After a 2-hour incubation, cells were washed, and color development was induced with NBT/BCIP. The assay results demonstrated strong binding of both DKK1-AP and DKK3-AP to cells expressing mLRPII and mLRPIV, with DKK3 also showing strong binding to mLRPIII (fig. S8A). Cell lysates were analyzed to quantitatively confirm these interactions (fig. S8, B to C). We further confirmed that both DKK1 and DKK3 robustly bound to the SH-SY5Y cell line stably expressing mLRPIV (mLRPIV^+^) (fig. S8D). These findings align with previous biochemical binding assays shown in Fig. 2, indicating that our cell-based AP-binding assay is suitable for high-throughput screening of inhibitory compounds.

Given the shared structural domains among DKK and LRP family members (*15*, *42*), we hypothesized that chemical inhibitors known to disrupt the DKK1-LRP6 interaction, specifically WAY262611 and IIIC3, might also inhibit the DKK-mLRPIV complex. We preincubated mLRPIV^+^ cells with graded doses of WAY262611 and IIIC3 for 2 hours, followed by the addition of DKK1-AP or DKK3-AP CM. Subsequent visual and quantitative AP activity assessments revealed that while 1 μM or higher concentrations of WAY262611 modestly reduced DKK1-mLRPIV binding, neither inhibitor affected the DKK3-mLRPIV interaction (fig. S8, E to G). These observations suggest the potential of DKK1-LRP6 inhibitors, or their chemical derivatives, to disrupt DKK1-LRP1 and possibly DKK3-LRP1 complex formation.

**Fig. 7.**
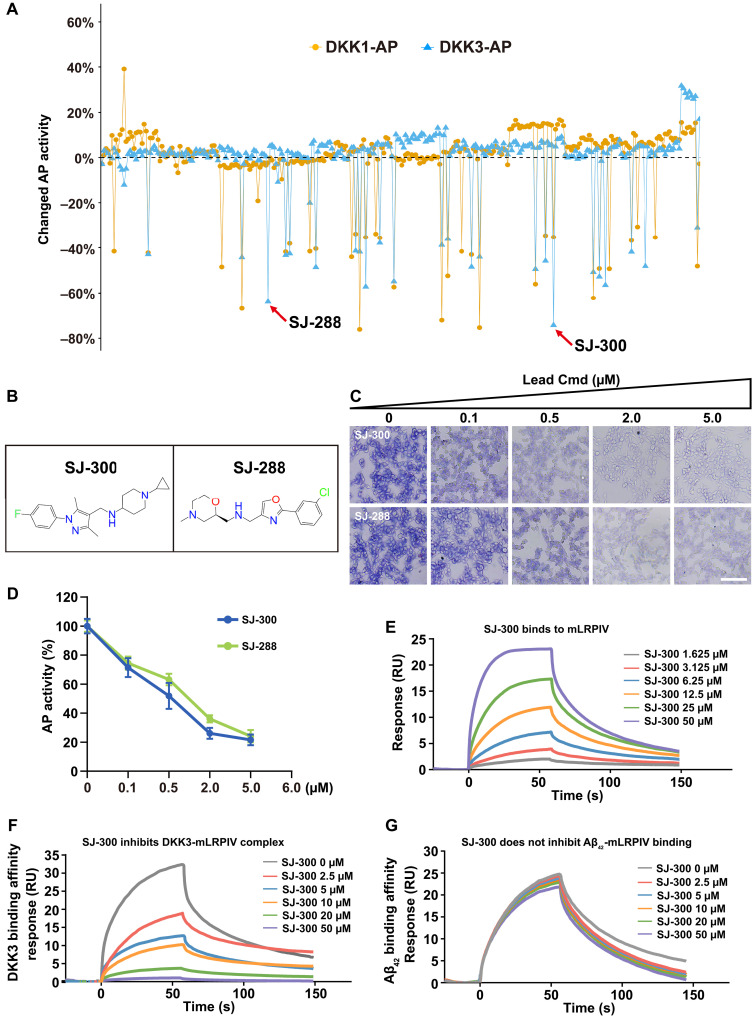
Screening for compounds that inhibit DKK-LRP1 complexes. (**A**) Quantitation of changes in AP activity in mLRPIV-expressing SH-SY5Y cells after treatment with 2 μM each of 300 selected compounds, followed by incubation with DKK1-AP CM or DKK3-AP CM. DMSO treatment is used as the baseline (0%). Red arrows highlight the two most potent inhibitors specific to the DKK3-LRP1 complex. (**B**) Chemical structures of the two lead inhibitors, named SJ-300 and SJ-288. (**C**) AP staining of cells treated with DKK3-AP CM containing varying dosages of SJ-300 or SJ-288. Cmd, compound. (**D**) Quantification of enzymatic AP activity from (C), normalized to DMSO treatment as 100%. Error bars represent mean ± SD from biological triplicates. (**E**) SPR analysis showing the binding affinity of SJ-300 (ranging from 1.625 to 50 μM) to immobilized mLRPIV (15 μM on the sensor chip). (**F**) Illustration of the inhibitory effect of SJ-300 on the DKK3-mLRPIV interaction, with response curves for various concentrations of SJ-300. A constant 35.5 nM DKK3 was used for incubation with mLRPIV. (**G**) SPR profile indicating that SJ-300 (0 to 50 μM) does not inhibit the binding of Aβ_42_ (1 μM fixed concentration) to mLRPIV.

Drawing upon the structural understanding of the DKK1-LRP6 binding interface and based on analogs of NCI8642 (IIIC3), which were originally characterized as inhibitors of the DKK1-LRP5 complex (*43*), we evaluated all chemicals in a library of 3000 potential DKK1 inhibitors from Enamine. Our goal was to select 300 compounds representing a diverse range of core structures for screening against DKK-mLRPIV complexes. Using the AP cell surface–binding assay, we cultured mLRPIV^+^ cells in 96-well plates. These cells were preincubated with 2 μM dimethyl sulfoxide (DMSO), WAY262611, or each of the 300 selected compounds for 2 hours, followed by the addition of DKK1-AP or DKK3-AP CM for an additional 2 hours. AP activity was then measured to assess binding. Notably, 28 of these compounds inhibited the DKK1-mLRPIV or DKK3-mLRPIV interaction by more than 40% (Fig. 7A).

Among the initial 28 candidates, we prioritized those that specifically inhibited the DKK3-mLRPIV interaction without affecting DKK1 binding. This strategic focus was designed to minimize potential side effects by not disturbing Wnt signaling pathways, thereby enhancing specificity for DKK3 and reducing the likelihood of adverse effects on Wnt complex roles in various organs. The efficacy of the two most potent compounds, designated SJ-288 and SJ-300 (Fig. 7, A and B), was further substantiated through dose-dependent studies. These studies demonstrated a substantial reduction in the DKK3-mLRPIV interaction (Fig. 7, C and D), confirming their potential as targeted therapeutic agents.

Together, our screening efforts have successfully identified compounds capable of inhibiting the DKK1-LRP1 and/or DKK3-LRP1 interactions. Among these, two standout candidates were found to selectively suppress the DKK3-LRP1 complex, highlighting their potential as therapeutic agents for conditions mediated by this interaction.

### Both SJ-288 and SJ-300 compounds effectively reverse the DKK3 inhibition of Aβ clearance

To assess the potential of SJ-288 and SJ-300 to facilitate Aβ clearance by inhibiting the DKK3-LRP1 interaction, we used the AD-NSC cell-based assay previously described (Fig. 3). After 14 days of differentiation, cells were treated as outlined in Fig. 3, with the addition of 1 μM of DMSO, SJ-288, or SJ-300 to the DKK1 or DKK3 CM. Fluorescent imaging demonstrated that both SJ-288 and SJ-300 effectively restored Aβ internalization in neuronal cells treated with DKK3 but not in those treated with DKK1 (fig. S9A). These findings were corroborated by ELISA assays, which indicated that SJ-288 and SJ-300 partially rescued Aβ uptake and subsequent clearance in DKK3-treated cells (fig. S9, B and C).

As a model test of whether the two DKK3 inhibitors affect the Wnt signaling pathway, the super 8 × TOPflash assays were used to assess Wnt activity as described (*22*). In SH-SY5Y cell cultures, as expected, DKK1 CM markedly inhibited Wnt3a-induced TOPflash activity, whereas DKK3 CM did not. Notably, at concentrations up to 10 μM, neither SJ-288 nor SJ-300 affected TOPflash activity when tested with DKK1 or DKK3 (fig. S9D). In addition, in the absence of DKK proteins, neither compound markedly altered Wnt activity at any tested concentration (fig. S9E). Thus, these data verify the efficacy of the two DKK3-specific inhibitors in augmenting Aβ uptake and clearance without directly affecting Wnt activity.

### Pharmacological analyses determine SJ-300 as a lead compound

To evaluate the metabolic stability of the candidate DKK3 inhibitors, we conducted microsomal stability assays. Notably, the concentration of SJ-300 remained stable after incubation with both mouse and human liver microsomes (fig. S10, A and B). In contrast, the stability of SJ-288 decreased sharply, with a marked reduction observed after 30 min and almost complete degradation by 60 min (fig. S10, C to D). These results suggest that SJ-300 has superior metabolic stability. Further assessing the in vivo stability of SJ-300, we administered the compound intravenously to C57 mice and measured its plasma levels at various time points up to 3 hours using liquid chromatography–tandem MS (LC-MS/MS). In agreement with in vitro microsomal results, SJ-300 demonstrated stable plasma concentrations throughout the time interval studied (fig. S10E).

To explore the potential of oral administration for chronic AD treatment, we assessed the bioavailability of SJ-300 in C57 mice using an established method (*44*). Following intragastric administration, SJ-300 was found at stable levels in plasma, similar to results observed with intravenous treatment (fig. S10F). Bioavailability calculations revealed that SJ-300 achieved a bioavailability of 71.3% (fig. S10G), which is promising for further drug development considerations. To determine the BBB penetration capabilities of SJ-300, we measured its brain/plasma (B/P) ratio over time. We found that this ratio gradually increased, reaching a notable level of 5732% at 6 hours postadministration (fig. S10H).

To confirm the direct binding of SJ-300 to LRP1, we carried out SPR experiments. The mLRPIV protein was immobilized on a CM5 sensor chip, and varying concentrations of SJ-300 were introduced to assess the binding interactions. The results demonstrated that SJ-300 effectively bound to mLRPIV, with a *K*_d_ of 7.9 μM (Fig. 7E). We also explored the potential of SJ-300 to inhibit the interaction between mLRPIV and DKK3. Different concentrations of SJ-300, combined with a fixed concentration of DKK3 (35.5 nM), were applied to the mLRPIV-bound sensor chip. The findings revealed that SJ-300 markedly impeded the binding of DKK3 to mLRPIV, with a median inhibitory concentration (IC_50_) value of 3.2 μM (Fig. 7F). Furthermore, unlike its inhibitory effect on the DKK3-mLRPIV complex, SJ-300 did not disrupt the binding of Aβ to LRP1 (Fig. 7G), reinforcing its specificity as an inhibitor of the DKK3-LRP1 interaction. Together, our results indicate that SJ-288 displays poor metabolic stability, whereas the pharmacodynamics and targeted inhibitory action of SJ-300 against the DKK3-LRP1 interaction support its further development as a promising candidate for AD therapy.

### SJ-300 effectively alleviates cognitive and pathological deficits in 5×FAD mice

**Fig. 8.**
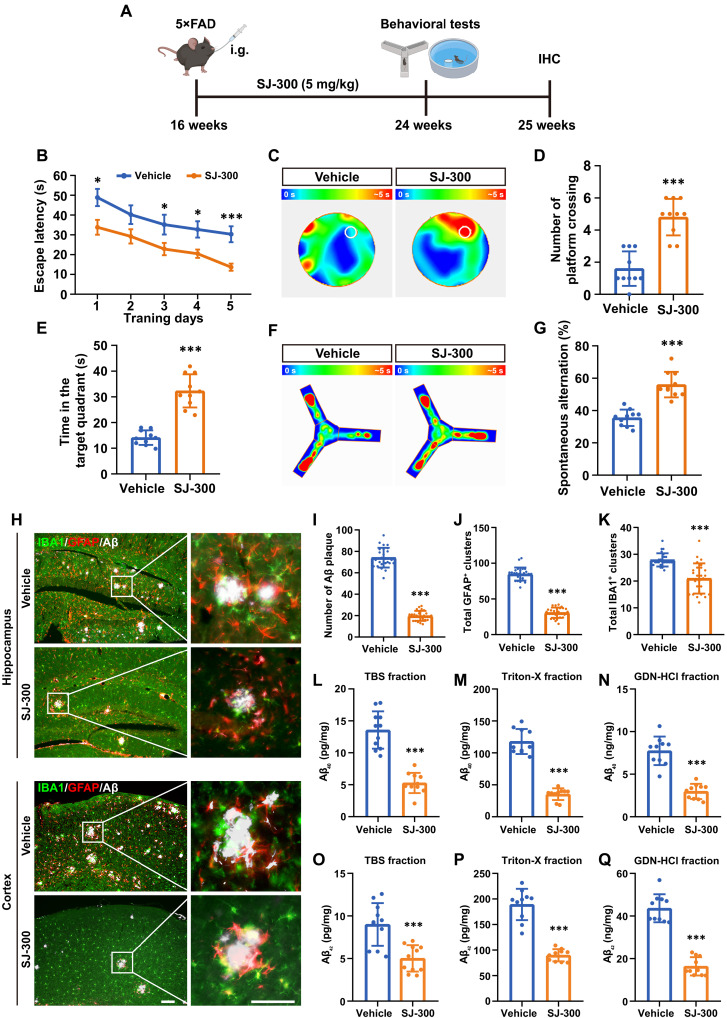
SJ-300 mitigates cognitive and pathological deficits of 5×FAD. (**A**) Schematic overview of the drug efficacy study design. 5×FAD mice, starting at 16 weeks old, were orally administered (intragastrically) with either vehicle or SJ-300 (5 mg/kg). Behavioral testing commenced at 24 weeks, followed by IHC staining. Each group consisted of *n* = 10 mice. Created in BioRender [R. Yang (2025); https://BioRender.com/b5jw7u3]. i.g., intragastrically. (**B**) Quantitation of the escape latency of mice from day 1 to 5 during MWM training. (**C**) Heatmap images from probe trials in MWM tests on day 6, with the platform location marked by a white circle. Quantitation showing the number of crossing the platform (**D**) and time spent (**E**) in the platform located quadrant. (**F**) Heatmaps showing the visit frequency of mice in Y-maze. (**G**) Quantification showing the percentage of spontaneous alternation in (F). (**H**) IHC staining for IBA1 (green), GFAP (red), and Aβ (white) in hippocampi and cortexes of mice treated with SJ-300 or vehicle. Enlarged images from white rectangles are displayed in the right panels. Scale bar, 50 μm. (**I** to **K**) Quantification of the number of Aβ plaque, GFAP^+^ clusters, and IBA1^+^ clusters per slice. (**L** to **Q**) ELISA measurements of Aβ_40_ (**L** to **N**) and Aβ_42_ (**O** to **Q**) in TBS, Triton-X, and guanidine fractions extracted from the brains of 6-month-old 5×FAD mice. Behavioral tests and Aβ ELISA assays included *n* = 10 mice per group; IHC assays analyzed *n* ≥ 30 slices randomly selected from at least 6 mice per group. Data are presented as mean ± SD. Statistical significance was assessed using the Student’s *t* test, with *P < 0.05 and ***P < 0.001.

To evaluate the therapeutic efficacy of SJ-300 in the 5×FAD transgenic mouse model of AD, we designed an experiment as outlined in the schematic diagram (Fig. 8A). Sixteen-week-old 5×FAD mice were divided into two groups and received intragastric injections once per day of either a vehicle solution or SJ-300 (5 mg/kg). After 8 weeks of treatment, cognitive functions were assessed using the MWM and Y-maze tests, along with histopathological analyses. In the MWM, SJ-300–treated mice demonstrated notable cognitive improvements as indicated by reduced escape latencies during the 5-day acquisition phase (Fig. 8B), increased time spent in the target quadrant, and higher numbers of platform crossings during the probe trials (Fig. 8, C to E). The Y-maze test further confirmed enhanced spatial working memory in the SJ-300 group, shown by increased spontaneous alternation rates (Fig. 8, F and G). In addition, control experiments conducted with WT mice showed that SJ-300 did not affect cognitive performance in a non-AD context (fig. S11).

To determine whether the cognitive improvements observed with SJ-300 treatment were associated with reductions in Aβ pathology, we examined histopathological changes in the cortex and hippocampus of euthanized mice. IHC revealed that SJ-300 treatment strongly reduced the number of Aβ plaques of various diameters in both brain regions (Fig. 8H). Quantitative analysis indicated a substantial decrease in the total number of Aβ plaques, ~73.3% (Fig. 8I). Corresponding with the reduced Aβ deposition, there was a notable decrease in the clustering of astrocytes and microglia around the plaques, with a reduction of about 64.1% in astrocytic cluster number (Fig. 8J) and considerable decreases in microglial clusters (Fig. 8K). Furthermore, SJ-300 markedly reduced both soluble and insoluble Aβ levels (Fig. 8, L to Q), mirroring the effects observed with DKK3 KO (Fig. 5). To explore whether SJ-300 influenced Aβ production, we used a stable HEK293 cell line expressing APP and presenilin 1 (PS1). After treating these cells with SJ-300, we assessed Aβ levels in the culture medium and APP cleavage–related proteins in cells via immunoblotting and ELISA. Results showed no change in the levels of β-site amyloid precursor protein-cleaving enzyme 1 (BACE1), PS1, and APP (fig. S12A), and similarly, Aβ_40_ and Aβ_42_ levels remained unchanged (fig. S12, B and C). Analysis of brain lysates from WT and 5×FAD mice also confirmed these findings (fig. S12D), suggesting that SJ-300 does not affect APP cleavage or Aβ production.

**Fig. 9.**
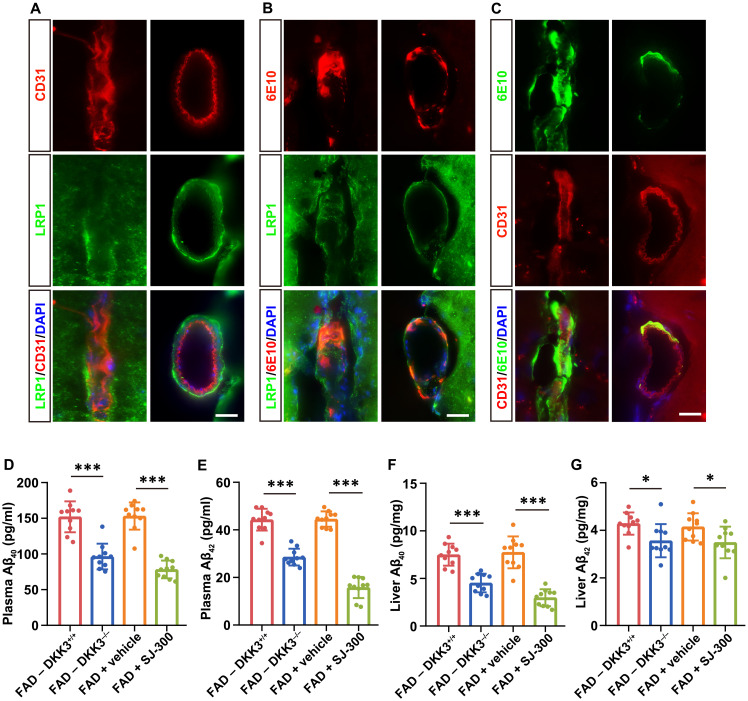
SJ-300 promotes peripheral clearance of Aβ in 5×FAD mice. (**A** to **C**) IHC images displaying the colocalization of Aβ and LRP1 in cerebral parenchymal (left panels) and meningeal (right panels) vessels in 6-month-old 5×FAD mice. CD31 is used as a marker for vascular endothelium. Scale bars, 20 μm. (**D** to **G**) ELISA depicting the levels of Aβ_40_ and Aβ_42_ in plasma [(D) and (E)] and liver [(F) and (G)] of 6-month-old 5×FAD mice under different genetic backgrounds or drug treatment conditions. Each ELISA assay included *n* = 10 mice. Data are presented as mean ± SD. Statistical significance was determined using the Student’s *t* test, with *P < 0.05 and ***P < 0.001.

Considering the pivotal function of LRP1, particularly soluble LRP1 (sLRP1), in the peripheral clearance of Aβ (*11*, *35*, *45*), we investigated the impact of SJ-300 on this process in 5×FAD mice aged 6 months. IHC analysis demonstrated LRP1 expression on the basement membrane of cerebral parenchymal and meningeal vessels, with Aβ localization observed on both the basement membrane and CD31-positive endothelium (Fig. 9, A to C). Subsequent ELISA measurements indicated that SJ-300 treatment, akin to DKK3-KO, reduced the levels of Aβ_40_ and Aβ_42_ in both plasma and liver of these mice (Fig. 9, D to G). In addition, we measured brain and plasma levels of sLRP1 via ELISA, finding that SJ-300 did not alter sLRP1 concentrations (fig. S13, A and B). To further explore the interaction between sLRP1 and DKK3, we conducted sLRP1 pull-down assays using these plasma samples. Results showed that SJ-300 substantially inhibited the association between sLRP1 and DKK3 (fig. S13C), corroborating its role in blocking the LRP1-DKK3 complex as previously demonstrated (Fig. 7). In sum, our findings demonstrate that SJ-300 treatment effectively attenuates cognitive deficits and AD pathologies, likely through enhancing Aβ clearance in AD model mice.

## DISCUSSION

Despite ongoing debate regarding the Aβ hypothesis, recent evidence continues to support the critical role of an imbalance between Aβ production and clearance in AD pathology (*46*, *47*). Human studies have now shown that defective Aβ clearance, rather than overproduction, drives Aβ accumulation and the pathogenesis of late-onset AD (*1*). The molecular mechanism of clearance is thus emerging as a promising target source for AD intervention. The clinical success of monoclonal anti-Aβ antibodies (mAbs) further highlights the importance of targeting Aβ clearance pathways as a strategy to modify the disease (*4*). Nonetheless, it is important to acknowledge certain limitations associated with recently approved Aβ immunotherapies, including limited effects on cognitive outcomes, incomplete targeting of soluble Aβ species, and the risk of amyloid-related imaging abnormalities (ARIA), which collectively highlight the need for alternative or complementary clearance-enhancing strategies. Our study identifies the DKK family proteins as previously unrecognized modulators of Aβ clearance, specifically through their interaction with LRP1, a key receptor involved in Aβ clearance. Further drug screening has also revealed the first chemical compound, in addition to mAbs, which promotes Aβ clearance.

Among molecules implicated in Aβ clearance, LRP1 has been most studied, owing to its abundant expression in the brain and involvement in several critical pathways (*48*, *49*). The latest studies have also identified LRP1 as a receptor for both tau and α-synuclein, two proteins that are crucial in the pathogenesis of neurodegeneration (*50*, *51*). Our findings confirm that LRP1 levels are lower in the human cortex of patients with AD compared to age-matched controls (Fig. 1, C and D), in accord with previous findings in a larger sample (*26*). To advance our understanding of AD pathology and facilitate drug screening efforts, we used multiple cell lines and NSC models derived from iPSCs of patients with AD to study LRP1-mediated Aβ clearance (Figs. 3 and 7 and fig. S9). Previously, DKK1, 2, and 4 were characterized as inhibitors of Wnt signaling, binding to the Wnt coreceptor LRP5/6. However, recent evidence suggests that DKKs may also exert Wnt-independent effects (*20*). In our study, we identified LRP1 as a membrane receptor for all DKKs, indicating that DKK-LRP1 complexes could affect LRP1 function in AD pathogenesis (Fig. 1). A soluble form of LRP1 (sLRP1), which can bind Aβ and facilitate its transport across the BBB, is critical for maintaining peripheral Aβ clearance (*34*). Our data suggest that DKKs interact with sLRP1, which retains all four ligand-binding domains (I to IV) present in FL LRP1 (Fig. 2 and fig. S13), although the precise effects of DKKs on sLRP1 function remain to be explored.

A promising clinical outcome for mAbs targeting the N-terminal region of Aβ is the reduction in PET-detectable insoluble Aβ, likely achieved by preventing Aβ aggregation and promoting its clearance. Notable reductions have been observed with gantenerumab (~100%), donanemab (~70%), and lecanemab (~90%) (*4*). However, the extent to which soluble Aβ species are reduced in clinical trials remains unclear. Our data show that DKK-LRP1 complexes play a critical role in mediating the endocytosis and clearance of soluble Aβ in both neurons and astrocytes, suggesting that DKKs and LRP1 may also be involved in mAb-induced Aβ clearance, potentially by regulating the dissolution of Aβ aggregates (*52*). Although the direct binding of Aβ to LRP1 is debated, our SPR data provide evidence for a direct interaction between monomeric Aβ and the IV ligand-binding domain of LRP1 (Fig. 4, A and C). However, the precise mechanism by which DKK and LRP1 influence mAb-induced Aβ clearance requires further investigation.

Our findings demonstrate that both DKK1 and DKK3 are up-regulated in patients with AD compared to age-matched healthy controls (Fig. 1, B and C), corroborating previous reports (*19*, *25*). While DKK1 function in AD has often been attributed to its Wnt-dependent effects (*53*), our data suggest a Wnt-independent mechanism by which DKK1 inhibits LRP1-mediated Aβ clearance. In contrast, the absence of appreciable Wnt-related effects in DKK3-KO mice, coupled with our findings that DKK3 binds strongly to LRP1, suggests that DKK3 may primarily affect Aβ clearance through LRP1 inhibition (Fig. 3 and fig. S5). This dual mechanism—blocking direct Aβ binding to LRP1 and promoting LRP1 endocytosis—could explain the role of DKKs in Aβ-mediated neurodegeneration, such as cell death (*19*) or synapse loss (*54*). DKK3 involvement in AD progression is supported by the results in 5×FAD mice, in which ablation of DKK3 showed reduction in Aβ pathology and improved cognitive function, while DKK3-OE worsened these outcomes (Figs. 5 and 6).

While DKK1 and DKK3 suppressed Aβ uptake in neurons and astrocytes, this effect was not observed in microglia (Fig. 3, E and F, and fig. S5E). This discrepancy likely reflects the distinct ligand-receptor landscape in microglia, which includes LRP1-associated inflammatory pathways (*30*, *31*), partial involvement of fluid-phase micropinocytosis (*32*), and potentially redundant or DKK-independent uptake mechanisms (*33*). Our in vitro assays were limited to soluble monomeric Aβ under basal conditions, whereas microglia in vivo are exposed to a diverse range of Aβ species—such as oligomers, fibrils, and plaques—which can engage different receptors and elicit varied cellular responses. Although DKK3 did not directly affect soluble Aβ uptake by microglia in vitro, we observed clear changes in microglial activation in both DKK3-deficient and SJ-300–treated AD mice (Figs. 5, 6, and 8). These phenotypes closely mirrored reductions in Aβ plaque burden, suggesting that the microglial changes may arise indirectly from diminished plaque pathology rather than from direct modulation of microglial phagocytosis. Given that plaque load and morphology strongly influence microglial activation states, a shift toward a less reactive phenotype is consistent with the observed decrease in IBA1^+^ microglial reactivity. Nevertheless, whether DKK3 modulates LRP1 signaling in microglia under pathological or plaque-associated conditions remains an open question. We are planning future studies using conditional genetic and pharmacological approaches to define the role of DKK-LRP1 interactions in microglial Aβ clearance, particularly within the complex in vivo environment.

To develop DKK-LRP1–targeted therapies, DKK3 stands out as a promising candidate due to its brain-enriched expression (Human Protein Atlas) and lack of involvement in Wnt signaling. High-throughput screening identified SJ-300 as a lead compound that selectively disrupts the DKK3-LRP1 interaction (Fig. 7 and fig. S8). SJ-300 treatment enhanced Aβ clearance and cognitive function in AD mice (Fig. 8), likely without affecting canonical Wnt signaling. Early pharmacokinetic and brain permeability data support its potential as a therapeutic candidate (fig. S10). However, its moderate in vitro IC_50_ underscores the need for further structure-activity optimization.

In conclusion, we identified DKK-LRP1 complexes as promising therapeutic targets for AD, providing the first chemical drug candidate for modulating Aβ clearance. Future research should concentrate on elucidating the detailed mechanisms by which DKK3 and SJ-300 influence brain efflux and peripheral metabolism of Aβ. Furthermore, comprehensive assessments of the structure-activity relationship (SAR) and long-term safety profile of the compound are essential, with a particular focus on ARIA as a potential side effect. Only if those studies are encouraging would preclinical research advance to detailed comparisons and even combinations of SJ-300 and anti-Aβ mAbs.

## MATERIALS AND METHODS

### Mice

Male 5×FAD mice (the Jackson Laboratory, stock #034848) were crossed with female DKK3^+^/^−^ heterozygotes (Cyagen, stock #KOCMP-50781-DKK3-B6N-VA) to obtain FAD-DKK3^−/−^ and FAD-DKK3^+^/^+^ mice. Sixteen-week-old 5×FAD mice (male:female ≈ 1:1) were randomly assigned to groups for AAV injection and SJ-300 drug delivery experiments. Mice received intragastric administration of SJ-300 (5 mg/kg; dissolved in 0.5% *O*-carboxymethylcellulose–Na in saline) or vehicle once daily for 8 weeks. Mice were housed in a temperature (26° to 28°C) and humidity-controlled environment with a 12-hour light-dark cycle. All procedures were approved by the Animal Ethics Committee of China Pharmaceutical University and complied with the Regulations for the Management of Laboratory Animals.

### Plasmids, antibodies, and cell lines

DKK4-Flag plasmid was constructed as described (*22*). Plasmids expressing DKK1-Flag, DKK2-Flag, DKK3-Flag, LRP6, 8 × TOPflash, and 8 × FOPflash were sourced from Addgene. DKK1-AP and DKK3-AP plasmids were generated by cloning the cDNAs of DKK1 and DKK3 into the pAPtag-5 vector (GenHunter, QV5), each fused with a C-terminal AP tag. Plasmids expressing the minireceptors mLRPI, mLRPII, mLRPIII, and mLRPIV were provided by G. Bu (Mayo Clinic). HEK293 cells transfected with the DKK4-Flag plasmid and SH-SY5Y cells transfected with the mLRPIV plasmid were selected using G418 (1 mg/ml; Invitrogen, 11811023) to establish stable cell lines. The cDNAs for human APP (hAPP) and human PS1 (hPS1), containing familial mutations, were amplified by PCR from genomic DNA isolated from 5×FAD mouse tails. These cDNAs, separated by a T2A motif, were cloned into the pHAGE-MCS-3×HA vector (MiaoLing, P23784). A stable HEK293 cell line expressing hAPP and hPS1 was subsequently created through a lentiviral transduction protocol developed by our research group. For the prokaryotic expression of mLRPIV, the cDNA was cloned into the pET24a vector with a C-terminal 6 × His tag to facilitate purification. Antibodies and commercial cell lines used in this study are detailed in table S1.

To generate LRP1-KO cell lines, single guide RNAs (sgRNAs) targeting human *LRP1* (5′-ATGCCAACGAGACCGTATGC-3′) and mouse *LRP1* (5′-CGGCTCGGGACCCCACTGAGGGG-3′) were cloned into the CRISPR-Cas9 expression vector pSpCas9(BB)-2A-Puro (PX459; Addgene, #101716). Lentiviral particles were produced by cotransfecting HEK293T cells with PX459-sgRNA constructs, psPAX2 (Addgene, #12260), and pMD2.G (Addgene, #12259) and collecting viral supernatants. Target cells were transduced and selected with puromycin (2 μg/ml) for 5 days, followed by a recovery period of 7 days in puromycin-free medium to establish LRP1-KO clones.

### Protein extraction from cultured cells

Cells were lysed using a lysis buffer containing 0.5% NP-40, 350 mM NaCl, 20 mM Hepes, and a protease inhibitor cocktail. Following lysis, samples were centrifuged at 2000 rpm for 5 min at 4°C to separate the cytoplasmic components. The supernatant was collected as the Cyt lysate. The pellet was washed twice with cold phosphate-buffered saline (PBS) and recentrifuged at 15,000 rpm for 10 min. The pellet was then resuspended in 5 M GDN-HCl, lysed, and centrifuged again at 15,000 rpm for 10 min. The resulting supernatant was transferred to a dialysis tube (Thermo Fisher Scientific, 69590) and dialyzed against PBS overnight at 4°C. The dialyzed solution was collected as the plasma Mem lysate. For the total cell lysate, cells were lysed in 2× sample buffer comprising 20 mM dithiothreitol, 6% SDS, 0.25 M tris (pH 6.8), 10% glycerol, 10 mM NaF, and bromophenol blue, at a concentration of ~1 × 10^7^ cells/ml. This mixture was heated in a boiling water bath for 5 min. Postheating, the lysates were sonicated for 10 s, repeated four times, and then centrifuged at 15,000 rpm for 10 min. The clear supernatant was collected as the total cell lysate.

### Gel filtration, immunoblotting, mass spectral analysis, IP, and gel silver staining

Superose 6 gel-filtration analyses were performed as described (*21*). Protein extracts (16 mg) were loaded onto the column preequilibrated with a buffer containing 20 mM Hepes (pH 7.9), 200 mM NaCl, 1 mM dithiothreitol (DTT), 0.1 mM phenylmethylsulfonyl fluoride (PMSF), and 10% glycerol. Fractions (1.5 ml) were collected and subjected to 10% SDS–polyacrylamide gel electrophoresis (SDS-PAGE), followed by immunoblotting. Band intensity was quantified using the ImageJ software. For IP, 1 ml of protein extract (8 mg/ml) was diluted 10-fold with IP buffer [20 mM Hepes (pH 7.9), 200 mM NaCl, 1 mM DTT, 0.2 mM PMSF, and 10% glycerol] and incubated with either 100 μl of anti-Flag M2 affinity gel (Sigma-Aldrich, A220) or 200 μg of anti-DKK4 antibody (R&D, MAB1269) and 100 μl rProtein A Sepharose gel (GE Healthcare, 17-1279-01) overnight at 4°C. Immunoprecipitates were washed four times with IP buffer and eluted with 100 mM glycine-HCl buffer (pH 2.5). The eluted proteins were then analyzed by immunoblotting. Less than 10% of total protein was used as an input control. Eluted proteins were prepared and shipped frozen on dry ice to the Taplin Biological Mass Spectrometry Facility at Harvard University for mass spectrometric analysis.

### Brain tissue homogenization and protein extraction

Human brain tissues were obtained from the Human Brain Bank of Central South University (ethical approval #: 2023-KT084) and from the National Institutes of Health (NIH) NeuroBioBank (protocol #: 918). Informed consent was obtained from the donors’ next of kin in accordance with institutional and national regulations. Human or mouse brain tissues were initially cut into small pieces in cold PBS and then homogenized using a Potter-Elvehjem tissue grinder (Thermo Fisher Scientific, 09-552-27). The dissociated cells were centrifuged at 3000 rpm for 15 min at 4°C and washed twice with cold PBS. The resulting cell pellets were resuspended in a lysis buffer [0.5% NP-40, 350 mM NaCl, and 20 mM Hepes (pH 8.0), enriched with a complete protease inhibitor cocktail] at three times the volume of the pellet. The suspension was mixed by pipetting 30 times and rotated at 4°C for 10 min. Following rotation, the lysate was centrifuged at 10,000 rpm for 15 min at 4°C. Glycerol was added to the supernatant to achieve a final concentration of 10% and stored at −80°C until further analysis. For separate protein extraction of brain tissue, TBS, Triton-X, and GDN-HCl were used to prepare lysates with modification (*40*). Initially, homogenized samples were centrifuged at ×100,000*g* for 60 min at 4°C. The supernatant was collected as the TBS fraction (soluble fraction). The pellet was then resuspended in 1% Triton-X with a protease inhibitor cocktail and incubated for 1 hour at 4°C. After centrifugation at the conditions mentioned above, the supernatant was collected as the 1% Triton-X fraction. The remaining pellet was further processed by rehomogenization in 5 M GDN-HCl (pH 7.6), incubated for 12 hours at 22°C, and centrifuged under the same conditions. The final supernatant (guanidine fraction) was diluted 10-fold with TBS. All lysates from each fraction were aliquoted and stored at −80°C until use.

### DAB and AP chromogen IHC

Paraffin-embedded human cortical sections were deparaffinized in xylene through three 10-min washes. This was followed by rehydration in a series of graded ethanol solutions (50, 70, 80, 90, and 100%) for 5 min each, concluding with a 10-min rinse in distilled water. Antigen retrieval was performed using sodium citrate buffer (VectorLabs, H-3300) under heated conditions to enhance antigen exposure. For 3,3′-diaminobenzidine (DAB) staining, sections were incubated with horseradish peroxidase (HRP)–conjugated secondary antibodies appropriate for the primary antibodies used. Visualization was achieved using a DAB chromogen kit (Beyotime, P0203), which results in a brown precipitate at the antigen site. DAB signal was measured and quantified using ImageJ. For green-color AP chromogen staining, sections were stained using an AP chromogen kit (Enzo, ENZ-ACC130-0030) according to the manufacturer’s instructions. This technique provides a distinct green coloration at the antigen sites, facilitating differential identification from DAB staining.

### In vitro protein synthesis

Proteins were synthesized using the T7 TNT Quick Coupled Transcription/Translation Kit, which uses a rabbit reticulocyte lysate system (Promega, L1170), following the manufacturer’s detailed instructions. The mLRPI, mLRPII, mLRPIII, and mLRPIV proteins were produced using plasmids encoding the respective minireceptors of LRP1 as templates. These plasmids were engineered to include sequences for a T7 promoter at the N terminus and an HA tag at the C terminus. These modifications were introduced via PCR amplification, adhering to the protocol provided by the kit manufacturer. PCR products were then purified using a DNA purification kit (QIAGEN, 28704). The synthesized proteins were purified on an HA affinity gel (Cell Signaling Technology, 3956), and their level was determined by immunoblotting with pure HA peptides (Sigma-Aldrich, 12149) as standards.

### Protein-protein binding, AP enzymatic activity, and SPR

The concentrations of DKK1-Flag and DKK3-Flag, as well as DKK1-AP and DKK3-AP (each also tagged with a single Myc epitope), in CM were quantified by immunoblotting using antibodies against Flag and Myc peptides (Sigma-Aldrich, F3290 and M2435, respectively). In some experiments, DKK proteins in CM were purified using anti-Flag gel or Ni-NTA Agarose beads (QIAGEN, 30210) to facilitate subsequent analyses. CM mixtures (1 ml) containing DKK3-Flag or DKK1/3-AP were incubated with various concentrations of purified mLRPI to IV and 20 μl of Sepharose beads for 4 hours at room temperature for IP and immunoblotting or for measuring AP activity. For the AP enzymatic activity assay, the beads were washed four times with PBS containing 0.1% Tween 20. After complete removal of the wash buffer, 100 μl of alkaline buffer with NBT/BCIP (Roche, 11681451001) was added, and the reaction was incubated for 15 min at room temperature. The reaction was stopped by adding 100 μl of 0.5 N NaOH, and absorbance at 650 nm was measured using a plate reader (PerkinElmer).

SPR experiments were conducted using a Biacore T200 system. Purified mLRPIV (15 μM) was covalently attached to a CM5 sensor chip using a standard amine coupling protocol. Measurements of protein-protein or protein-compound affinities were performed with various concentrations of DKK1 or DKK3 (6.25, 12.5, 25, 50, 100, and 200 nM), and SJ-300 (1.625, 3.125, 6.25, 12.5, 35, and 50 μM). Competitive binding studies included varying concentrations of Aβ_42_ (0, 100, 200, 300, 400, and 500 nM) or SJ-300 (0, 2.5, 5, 10, 20, and 50 μM). The SPR data analysis, facilitated by Biacore T200 software, provided the association rate constant (*K*_a_) and equilibrium dissociation rate constant (*K*_d_).

### Cell culture and CM preparation

293, SH-SY5Y, C8-D1A (astrocytic), SIM-A9 (microglial), and PEA-13 (LRP1-deficient) cell lines were cultured in Dulbecco’s Modified Eagle Medium (DMEM) supplemented with 10% fetal bovine serum (FBS) at 37°C in a 5% CO_2_ atmosphere. Human iPSC-derived NSCs (Axol Bioscience, ax0111) were maintained according to the manufacturer’s protocols on Laminin-coated chamber slides (Thermo Fisher Scientific, 154526). These cells were cultured in Neural Expansion Medium (Axol Bioscience, ax0030500) supplemented with EGF (20 ng/ml) and fibroblast growth factor 2 (FGF2; 20 ng/ml). For differentiation, NSCs were cultured in medium without EGF and FGF2 for 14 days before experiments. For CM collection, HEK293 cells (2 × 10^8^) were transfected with 100 μg of DKK1-Flag, DKK3-Flag, DKK1-AP, or DKK3-AP plasmids using Lipofectamine 2000 (Invitrogen) according to the manufacturer’s instructions. Following a 16-hour posttransfection period, the culture media were replaced with DMEM containing 1% FBS to collect CM. After a 24-hour incubation period, the media were concentrated at least 10-fold using Centricon Plus filters (Millipore, UFC9010) and subsequently stored at −80°C until needed. Media from untransfected HEK293 cells served as the control CM. In subsequent experiments, cells were treated with a 1:1 mixture of CM and fresh culture medium. For some assays, recombinant DKK1 and DKK3 proteins (R&D Systems, #5439-DK and #1118-DK) were used directly. In other cases, DKK proteins were purified from CM using anti-Flag affinity gel to ensure high purity for subsequent biochemical and cellular analyses.

### Prokaryotic expression and purification

*Escherichia coli* BL21 (DE3) cells were cultured, and protein expression was induced with 1 mM isopropyl-β-d-thiogalactopyranoside at 37°C for 12 hours. Cells were then harvested by centrifugation at room temperature for 10 min (15,000 rpm) and resuspended in lysis buffer [50 mM NaH_2_PO_4_, 300 mM NaCl, and 10 mM imidazole (pH 8.0)], followed by sonication and centrifugation at 4°C for 30 min (15,000 rpm). The supernatants containing 6 × His–tagged proteins were applied to Ni-NTA Agarose beads preequilibrated with lysis buffer. To remove nonspecific bound proteins, the agarose beads were washed with buffer [50 mM NaH_2_PO_4_, 300 mM NaCl, and 20 mM imidazole (pH 8.0)], and the His-tagged proteins were then eluted using elution buffer [50 mM NaH_2_PO_4_, 300 mM NaCl, and 250 mM imidazole (pH 8.0)]. The purity and identity of proteins were confirmed by SDS-PAGE, followed by Coomassie Brilliant Blue staining using a kit (Bio-Rad, 1610435).

### Plasma and liver sample preparation

Following the completion of behavioral tests, mice were euthanized, and blood samples were immediately collected via cardiac puncture. Plasma was obtained by centrifuging the blood at 4,000 rpm for 15 min at 4°C. The separated plasma was then aliquoted and stored at −80°C for analyses. Livers were removed and homogenized in radioimmunoprecipitation assay buffer (Thermo Fisher Scientific, 89900) at a concentration of 1:10 (g/v) with 1% PMSF. Supernatant proteins were extracted after centrifugation at 13,000 rpm for 20 min at 4°C. For each sample, 100 μl of extracted protein was used for ELISA measurement.

### Aβ uptake, clearance assay, and Aβ ELISA

Aβ_42_ and 5(6)-FAM–Aβ_42_ (AnaSpec, AS20276 and AS2352601) were pretreated with trifluoroacetic acid and 2-propanol as described (*28*) and stored at −80°C. Aβ peptides were freshly dissolved in DMSO at 200 μM until use. For the Aβ_42_ uptake assay, cells were incubated with 1 μM Aβ_42_ for 24 hours. Following incubation, cells were lysed in 5 M GDN-HCl (in 50 mM tris-HCl, pH 8.0) to extract Aβ_42_. The concentration of cellular Aβ_42_ was quantified using a human Aβ_42_ ELISA kit (Invitrogen, KHB3441) following the manufacturer’s instructions. For Aβ_42_ clearance calculation, cells were preincubated with CM for 2 hours before adding Aβ_42_ to the cultures for an additional 2 hours. The Aβ-containing medium was then replaced with fresh medium devoid of CM or Aβ_42_, and the cells were incubated for another 8 hours. Cell-associated Aβ_42_ was measured by ELISA to determine the clearance rate, calculated as the reduction in Aβ_42_ levels after the 8-hour incubation. The reduced amount of Aβ_42_ after the 8-hour incubation was determined as Aβ_42_ clearance. Levels of Aβ_40_ and Aβ_42_ in brain tissue (three fractions), plasma, and liver were determined using specific human ELISA kits (Invitrogen, KHB3481 for Aβ_40_ and KHB3441 for Aβ_42_), according to the manufacturer’s protocols.

### ELISA for sLRP1 and sLRP1-associated DKK3

Brain and plasma sLRP1 levels were determined by sLRP1 ELISA with modification (*36*). ELISA plates (Nunc, 446469) were coated overnight at 4°C with 1 μg per well of RAP (Invitrogen, RP-102336). Plates were then blocked with Hanks’ balanced salt solution (HBSS) buffer (20 mM Hepes, 2 mM CaCl_2_, 1% bovine serum albumin, and 0.1% Tween 20) for 30 min at 37°C. Diluted plasma samples, brain soluble fractions, or human plasma–derived sLRP1 standards (100-fold dilution in blocking buffer) were added to each well and incubated for 1 hour at room temperature. After extensive washing with HBSS, wells were incubated with a human-specific LRP1 8G1 antibody (Abcam, ab20384), followed by an HRP-conjugated goat anti-mouse secondary antibody (Jackson ImmunoResearch, 115-035-003) at a 1:5000 dilution for 1 hour at room temperature. For color development, 3,3′,5,5′-tetramethylbenzidine (Sigma-Aldrich, 860336) was added, and the reaction was stopped with 1 N HCl. Absorbance was measured at 450 nm using a PerkinElmer plate reader, and sLRP1 concentrations were calculated from a standard curve.

To assess levels of sLRP1-associated DKK3, plasma samples were incubated overnight at 4°C with 4 μg of anti-LRP1 antibody (Abcam, ab92544) in the presence of Protein G agarose (Roche, 11243233001). The agarose beads were collected by centrifugation, washed as per the Protein G IP kit instructions (Roche, IP50-1KT), and then resuspended in 10 μl of 5 M GDN-HCl/50 mM tris-HCl (pH 8.0). This mixture was incubated for 4 hours at room temperature and diluted 50 times, and the levels of sLRP1-bound DKK3 were determined using an anti-DKK3 antibody (Abcam, ab186409) in an ELISA setup as described previously.

### Fluorescence, IF, and microscopy

Cells were cultured in Lab-Tek II chamber slides (Thermo Fisher Scientific, 154526) at 37°C for at least 24 hours before experiments. Before the addition of FAM-Aβ_42_ (1 μM) at 37°C, cells were preincubated with or without CM for 2 hours. After 24 hours, cells were fixed using 4% paraformaldehyde (PFA), and the fluorescence-labeled Aβ was observed by Delta Vision microscopy (GE Healthcare). LysoTracker Red (50 nM; Molecular Probes) was added to the medium 30 min before fixation. Postfixation, Alexa Fluor 594 Phalloidin (1:100) was applied. For IF, cells were fixed and immunostained with anti-Tuj1, anti-nestin, or anti-GFAP antibody. For cryostat sectioning of brain tissues, 20-μm frozen slices were sectioned by a Leica CM1950 microtome and followed a protocol developed in our group. Fluorescence images were captured using a fluorescence microscope (Olympus, IX73). Antibodies used were listed in table S1.

### Cell surface–binding assay

HEK293 or SH-SY5Y cells were cultured on 0.2% gelatin-coated coverslips or in 96-well plates. AP binding assays were performed using a kit (GenHunter, Q502) with modifications. CM containing AP, DKK1-AP, or DKK3-AP was added to cells with or without preincubation of compounds such as WAY262611 and IIIC3 (Santa Cruz Biotechnology, sc-397019 and sc-397019) or other compounds selected from a commercial library (Enamine). After 2 hours of incubation at 37°C, cells were washed four times with warm PBS. For cell imaging, cells were fixed with 4% PFA for 2 min and then washed, and the NBT/BCIP substrate (Roche, 11681451001) was added for color development. For quantitative AP enzyme activity measurement, cells cultured in a 96-well plate were lysed with 100 μl of cell lysis buffer (GenHunter, Q504) for 5 min, followed by shaking for 10 s and removing cell nuclei by a 2-min centrifugation at 1000 rpm. Supernatants of cell lysates were removed into another 96-well plate and incubated at 65°C for 10 min to inactivate endogenous AP activity. A total of 10 μl of cell lysate was added to 90 μl of reaction buffer [0.1 M tris-HCl (pH 9.5) and 0.1 M NaCl] with NBT/BCIP substrate for 15 min. The reaction was stopped by adding 100 μl 0.5 N NaOH and absorption measured at 650 nm using a Victor plate reader (PerkinElmer).

### Top/FOP luciferase reporter assay

The 8 × Top/FOP luciferase reporter system (*22*) was used to measure Wnt/β-catenin activity. HEK293 cells (1 × 10^6^/500 μl of DMEM) were transiently transfected with 100 ng of constitutively active vector encoding *Renilla* luciferase (Promega, E2231) for readout of activity and 1 μg of TOPflash or negative control FOPflash using the Nucleofector system (Lonza). After 24 hours of transfection, cells were incubated with compounds only or compounds along with DKK1 CM or DKK3 CM for 2 hours, followed by the addition of Wnt3a (50 ng/ml) to the cultures for 24 hours. Firefly and *Renilla* luciferase activity were then measured using a dual-luciferase kit (Promega, E1910) according to the manufacturer’s instructions. The firefly luciferase activity was normalized to *Renilla* luciferase activity, and the fold increase in TOPflash activity compared to FOPflash is reported.

### Preparation of AAV

The pAAV-CAG-EGFP plasmid (Addgene, 37825) was modified to include a T2A self-cleaving peptide and a multiple cloning site (MCS) downstream of the EGFP gene. Human DKK3 cDNA was then cloned into the MCS using HindIII and NheI restriction sites. The detailed structures of the AAV plasmids used in this study are depicted in fig. S6. AAV was produced in HEK293 cells following a standardized protocol available from Addgene (www.addgene.org/protocols/aav-production-hek293-cells/). Briefly, HEK293 cells were transfected with the recombinant AAV plasmid along with helper plasmids to facilitate AAV replication and packaging. Posttransfection, cells were harvested, and the virus was extracted from the cell lysates and purified through iodixanol gradient ultracentrifugation. The purified viral particles were then titrated and stored at −80°C until further use.

### Brain stereotaxic injection

Brain stereotaxic injection was performed using our standard protocols. Mice were deeply anesthetized with an intraperitoneal injection of 1% pentobarbital (50 mg/kg). For AAV injection, 2 μl of AAV particles (~1 × 10^13^ viral genomes (v.g.)/ml) were bilaterally injected into the hippocampal CA1 region at −2.2 mm from bregma, mediolateral 1.7 mm, depth 2.4 mm of mouse brains. The injection process was conducted at a rate of 0.25 μl/min using a digital pump, maintaining the syringe in place for 10 min postinjection before slowly withdrawing it automatically. All mice were placed on a heating pad until they recovered from the surgery.

### MWM and Y-maze

The MWM was performed following our previously protocol. Mice were trained to locate a hidden escape platform (10 cm in diameter and 1 cm below the water surface) in a circular pool (130 cm in diameter and 60 cm deep) filled with warm water (25° ± 1°C). Training spanned five consecutive days, with four trials/day. Each trial began at the same time each day. Mice were released from different starting positions (north, east, southeast, and northwest) facing the pool wall in a different order each day. They were allowed 1 min to find the hidden platform. If a mouse did not find the platform within this time, then it was guided to it by the experimenter. Once on the platform, the mouse remained there for 15 s to memorize spatial cues before being placed back in its cage for a 15-s rest until next trial. The location of the platform was kept constant throughout the experiment. On the sixth day, probe trials were conducted by removing the platform and placing the mice in the opposite sector, videotracking their movements for 1 min.

Y-maze experiments were conducted as described before. The Y-maze apparatus was made of black acrylic plastic, with each arm positioned at 120° angles to the others. The floor was 4 cm wide, each arm was 36 cm long, and the walls were 15.5 cm high. A camera placed directly above the maze recorded behavioral data, and ANY-maze software was used for tracking and analysis. Before the behavioral tests, the animals acclimated in the test chamber for at least 30 min in a quiet and stress-free environment. Each mouse was placed in the Y-maze for ~8 min. After the test, mice were carefully removed from the maze. The maze was cleaned with 75% ethanol and distilled water after each test and allowed to dry completely to eliminate any residual odor or moisture. A 5-min interval was maintained between tests to ensure the maze reached a neutral odor baseline. All experimental conditions, including light, noise levels, and temperature, were kept consistent between group-reared controls and socially isolated mice, ensuring that any behavioral differences were due solely to the social condition.

### In vitro metabolic analysis using liver microsomes

Human liver microsomes (from 50 donors) and CD1 mouse liver microsomes were acquired from Thermo Fisher Scientific (#HMMCPL and #MSMCPL). Microsomes were thawed slowly on ice and adjusted to a concentration of 20 mg/ml. Each incubation system had a total volume of 200 μl, comprising 183 μl of 100 mM PBS (pH 7.4), 2 μl of 50 μM SJ-300 or SJ-288, and 5 μl of microsomes (20 mg/ml). The microsomes, buffer, and test compounds were preincubated in a water bath at 37°C for 5 min. The reaction was initiated by adding 10 μl of 20 mM reduced form of nicotinamide adenine dinucleotide phosphate (NADPH) [containing 1 mM NADP, 5 mM glucose 6-phosphate, glucose 6-phosphate dehydrogenase (1 U/ml), and 3.3 mM MgCl_2_]. Incubation was continued at 37°C in a mild water bath, with each sample processed in triplicate. Samples lacking the NADPH generating system served as controls. The reaction was terminated at 0, 30, and 60 min by adding 200 μl of ice-cold methanol containing 2 μM of the internal standard, ornidazole. Samples were vigorously vortexed and subsequently centrifuged at 10,000 rpm for 10 min at 4°C. The clear supernatant was collected for analysis. A 2-μl aliquot of the supernatant was injected into an ultraperformance LC–MS/MS (UPLC-MS/MS) system to quantify the metabolic stability of the test compounds.

### Plasma metabolic analysis of SJ-300

Twelve male C57 mice were fasted for 12 hours and then randomly divided into two groups for the administration of SJ-300. The compound was delivered either intravenously at a dose of 2 mg/kg or intragastrically at 10 mg/kg. Blood samples (0.5 ml each) were collected from the orbital venous plexus at predetermined time intervals (0, 10, 30, 60, 120, and 180 min) postadministration. Collected blood was immediately transferred into 1.5-ml tubes containing EDTA as an anticoagulant. Samples were then centrifuged at 5000 rpm for 10 min at 4°C. A 100-μl aliquot of the resultant plasma was transferred into new 1.5-ml Eppendorf (EP) tubes and stored at −80°C until further processing. Upon thawing the plasma samples at 4°C, 25 μl of 50% methanol aqueous solution, 10 μl of 1% formic acid aqueous solution, 20 μl of geranylgeranyl (100 ng/ml), and 400 μl of acetonitrile were added. The mixture was vortexed for 5 min, ultrasonicated for 10 min, and frozen at −20°C for 30 min. After centrifugation at 4°C for 10 min (14,000 rpm), the supernatant was evaporated to dryness under nitrogen gas. The residue was reconstituted with 200 μl of 50% methanol by ultrasonication, centrifuged at 4°C for 10 min (14,000 rpm), and the supernatant used for UPLC-MS/MS analysis.

### Drug bioavailability analysis

To evaluate the bioavailability of SJ-300, nine male C57 were randomly divided into two groups. After a 12-hour fasting of the mice, SJ-300 was administered either intravenously (2 mg/kg) or intragastrically (10 mg/kg). Blood samples (0.5 ml) were collected from the orbital region at various intervals (0, 0.083, 0.25, 0.5, 1, 2, 4, 6, 8, 10, and 24 hours). The plasma was resolubilized with methanol, centrifuged at 4°C for 10 min (10,000 rpm), and the supernatant used for UPLC-MS/MS analysis. The pharmacokinetic data, including maximum blood concentration (*C*_max_), time to peak (*T*_max_), half-life (*t*_1/2_), total area under the curve, relative bioavailability (*F*), and mean residence time were analyzed using the DAS2.0 software.

### Brain penetration of SJ-300

The extent of drug transport across the BBB is characterized by the B/P ratio. SJ-300 was administered to 12 male C57 mice by intragastric injection (10 mg/kg), and blood samples were collected at different intervals (0.5, 1, 4, and 6 hours). Mice were then perfused with ice-cold PBS, and brain tissues were carefully removed. The brain samples were weighed and homogenized in ice-cold PBS [1:6 (w/v)] at −10°C for 30 s. Homogenized samples (100 μl) were placed into 1.5-ml polypropylene tubes, and 10 μl of inosine 5ylene tubes.510 (5 μg/ml) were added and mixed well. Ethyl acetate (1.0 ml) was added and mixed, and the mixture was centrifuged at 4°C for 5 min (12,000*g*). The supernatant (0.9 ml) was transferred to a new EP tube and dried by evaporation with a gentle stream of nitrogen in a 35°C water bath. The dried residue was resuspended in 100 μl of 80% methanol, rotated, stirred for 2 min, and centrifuged at 4°C for 10 min (22,000*g*), and 10 μl of the supernatant were injected into the LC-MS/MS system for quantitative analysis.

### Data and statistical analyses

All data were expressed as mean ± SD. Student’s *t* test was used to identify statistically significant differences between groups. GraphPad Prism 8.0 was used for statistical analysis. *P* < 0.05 was considered statistically significant.
