# Supplementary material for: DKK3-LRP1 complex and a chemical inhibitor regulate Aβ clearance in models of Alzheimer’s disease
Source: Sci Adv. 2025 Nov 7;11(45):eadz2099. doi: 10.1126/sciadv.adz2099 (PMC12594171; doi:10.1126/sciadv.adz2099)
Supplement: Supplementary file 1 — Figs. S1 to S13 Tables S1 and S2 [file sciadv.adz2099_sm.pdf]

Science Advances
AAAS

Supplementary Materials for

DKK3-LRP1 complex and a chemical inhibitor regulate Aβ clearance in models of Alzheimer's disease

Ruihan Yang *et al.*

Corresponding author: Jian Sima, simajian@cpu.edu.cn

Sci. Adv. **11**, eadz2099 (2025)
DOI: 10.1126/sciadv.adz2099

This PDF file includes:

Figs. S1 to S13
Tables S1 and S2

Fig. S1.

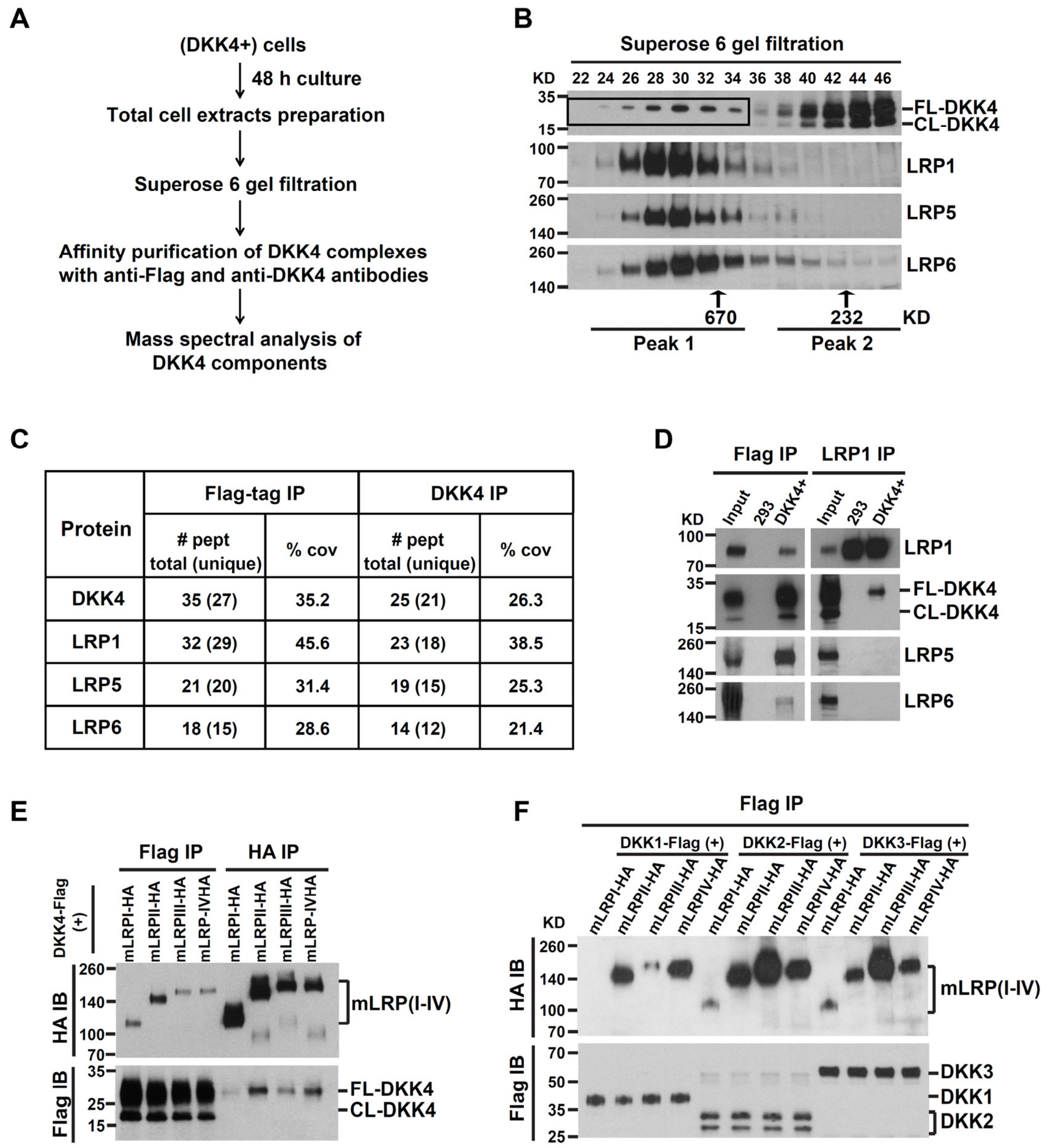

Protein	Flag-tag IP		DKK4 IP	
	# pept total (unique)	% cov	# pept total (unique)	% cov
DKK4	35 (27)	35.2	25 (21)	26.3
LRP1	32 (29)	45.6	23 (18)	38.5
LRP5	21 (20)	31.4	19 (15)	25.3
LRP6	18 (15)	28.6	14 (12)	21.4

Supplementary Fig. S1 Affinity purification of protein complexes reveals DKK binding to LRP1

(A) Schematic representation of the workflow for the purification of DKK4 protein complexes. **(B)** Immunoblot (IB) analysis displaying gel filtration profiles of fractionated extracts from DKK4-expressing cells. A rectangle indicates the fractions that were pooled for subsequent IP-Mass Spectral (MS) analysis. FL denotes full-length proteins; CL denotes cleaved proteins. **(C)** Mass spectrometry (MS) analysis detailing the components of the DKK4-LRP1 and DKK4-

LRP5/6 complexes isolated from DKK4-expressing cells. The data include the number of peptides (# pept), total peptides identified (total), and unique peptides (unique) characterizing each complex. **(D)** Immunoprecipitation (IP) using anti-Flag (left) or anti-LRP1 (right) antibodies performed on extracts from DKK4-expressing cells, followed by IB to detect specific protein interactions with the indicated antibodies. **(E)** Analysis of DKK4-expressing cell extracts transfected with vectors expressing HA-tagged mini-LRP1 receptors (mLRPI, mLRPII, mLRPIII, mLRPIV). Extracts were immunoprecipitated using anti-Flag or anti-HA antibodies, followed by IB with corresponding antibodies to verify the specificity of interactions. **(F)** Lysates from 293 cells co-transfected with plasmids expressing mini-LRP1 receptors (mLRPI, mLRPII, mLRPIII, mLRPIV) and constructs for DKK1-Flag, DKK2-Flag, or DKK3-Flag. The cell lysates were subjected to IP using anti-Flag antibody and analyzed by IB to explore binding affinities and specificity among the proteins.

Fig. S2.

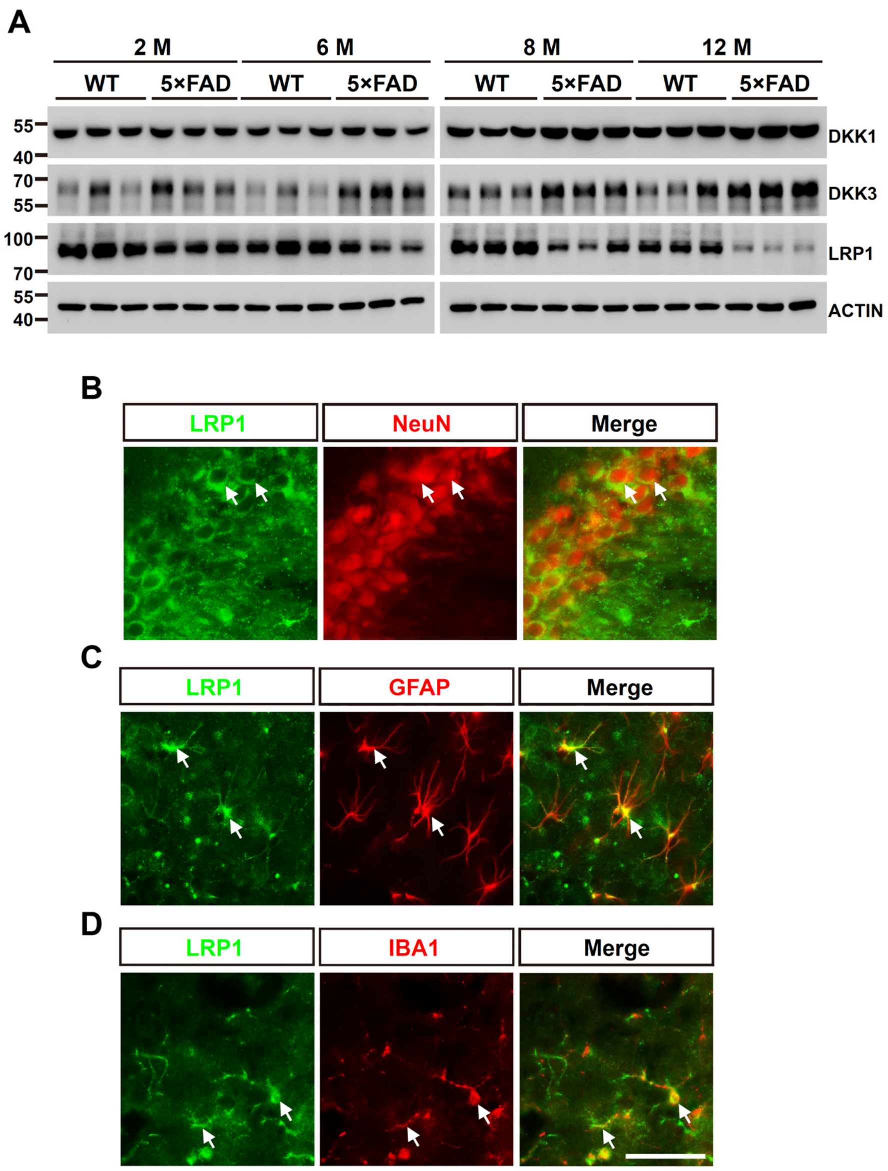

Supplementary Fig. S2 The expression pattern of DKK1 and DKK3 in mouse hippocampus **(A)** Immunoblot analysis of DKK1, DKK3, and LRP1 in hippocampal lysates from additional 3 wild-type (WT) and 5×FAD mice aged 2 to 12 months. **(B–D)** Representative immunohistochemical staining of LRP1 (green) co-localized with NeuN (**B**, red), GFAP (**C**, red), or IBA1 (**D**, red) in the hippocampus of 6-month-old WT mice, showing expression in neurons, astrocytes, and microglia, respectively. Arrows indicate cells exhibiting LRP1 co-localization. Scale bar, 50 μm.

Fig. S3.

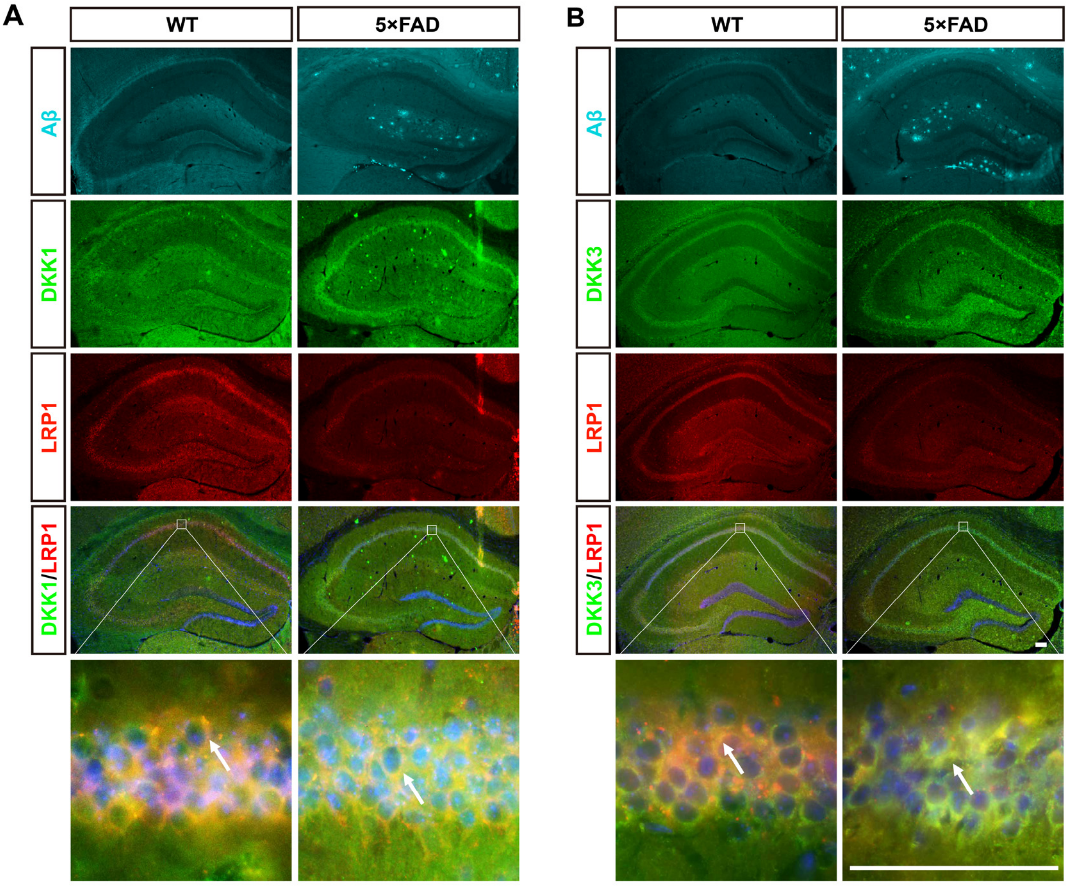

Supplementary Fig. S3 Co-localization of DKK1 and DKK3 with LRP1 in Mouse Hippocampus

(A) Immunohistochemistry (IHC) staining depicting the localization of amyloid-beta (Aβ) in blue, DKK1 in green, and LRP1 in red within the hippocampal regions of wild-type (WT) and 5×FAD mice. Enlarged images from the areas highlighted by white rectangles are shown in the lower panels to emphasize the detailed cellular distribution. Arrows indicate cells exhibiting co-localization of LRP1 with DKK1. **(B)** Similar to (A), but displaying the staining for DKK3 instead of DKK1. Arrows point to cells where LRP1 and DKK3 co-localize. The scale bar represents 200 μm.

Fig. S4.

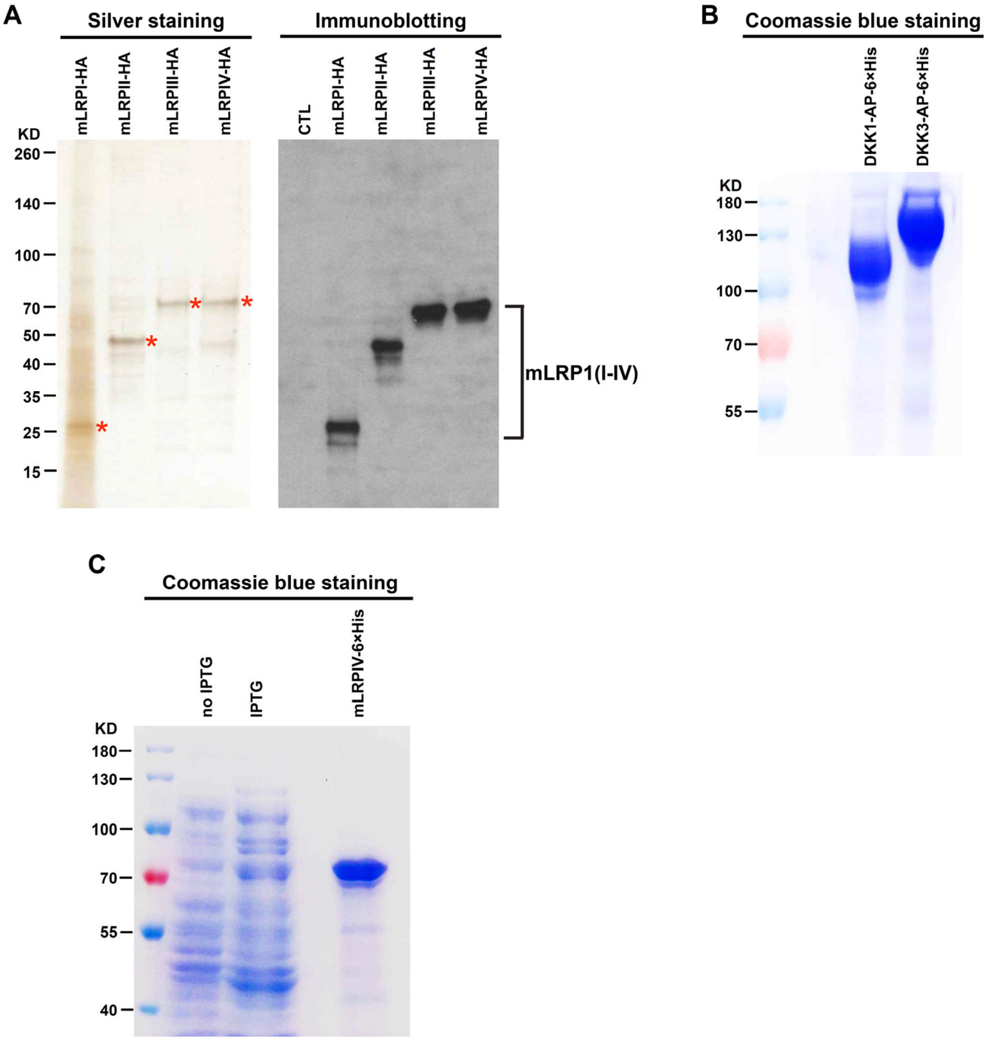

Supplementary Fig. S4 *In vitro* protein synthesis and purification of mLRPI-IV and DKKs

(A) Visualization of mLRPI-IV proteins synthesized using the rabbit reticulocyte lysate system. The left panel shows a silver-stained gel of the synthesized mini-LRP1 receptors (mLRPI, mLRPII, mLRPIII, and mLRPIV) marked by expected molecular weights (indicated with red stars). The right panel presents an immunoblot analysis of these proteins, demonstrating their successful immunopurification. Protein synthesis buffer without cDNA served as the control (CTL). **(B, C)** Coomassie Brilliant Blue-stained polyacrylamide gels displaying the synthesized and purified DKK1-AP and DKK3-AP proteins alongside mLRPIV. These panels highlight the amount and purity of each protein, confirming the effectiveness of the synthesis and purification processes.

Fig. S5.

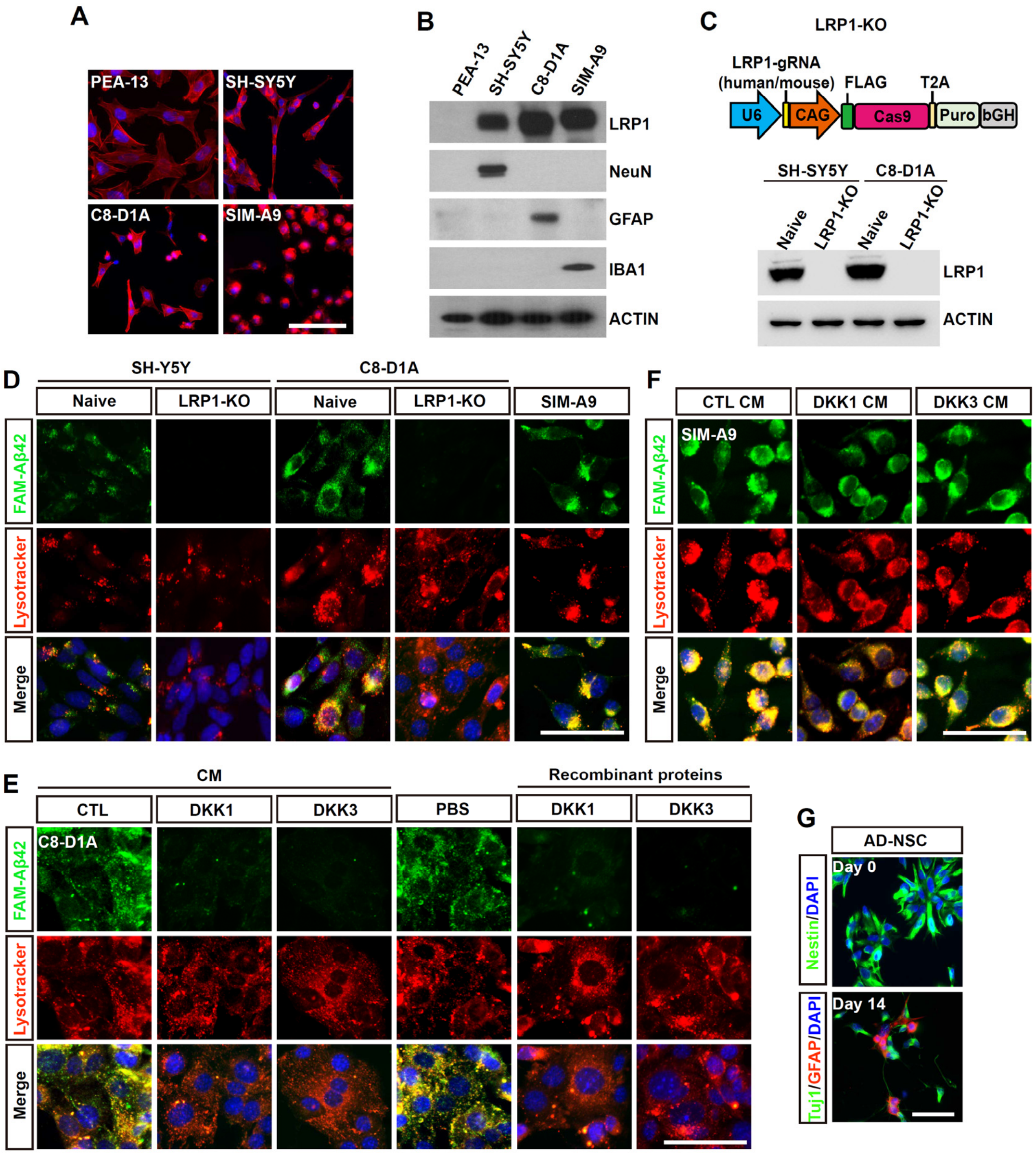

Supplementary Fig. S5 Examining Aβ endocytosis in cell-based assays

(**A**) Images of the morphology of each type of cells with Alexa Fluor® 594 Phalloidin staining (red). (**B**) IB showing the expression of LRP1, NeuN, GFAP, and IBA1 in each cell line. (**C**) Schematic of lentiviral vectors used to generate stable LRP1-KO cell lines. Immunoblotting showing the LRP1 levels in CTL and LRP1-KO cells. (**D**) Subcellular location of internalized FAM-Aβ42 (green) co-localized with lysosomal marker Lysotracker (red) in each indicated cell line. (**E**) Subcellular co-localization of FAM-Aβ42 (green) with Lysotracker (red) in C8-D1A cells

following treatment with DKK CM or recombinant proteins. (**F**) As in (D), except using SIM-A9 cells treated with indicated CM. (**G**) IHC images of the staining of Nestin at Day 0, or staining of Tuj1 and GFAP at Day 14 in AD-NSCs. Cell nuclei counterstained with DAPI (blue). Scale bars, 50 μm.

Fig. S6.

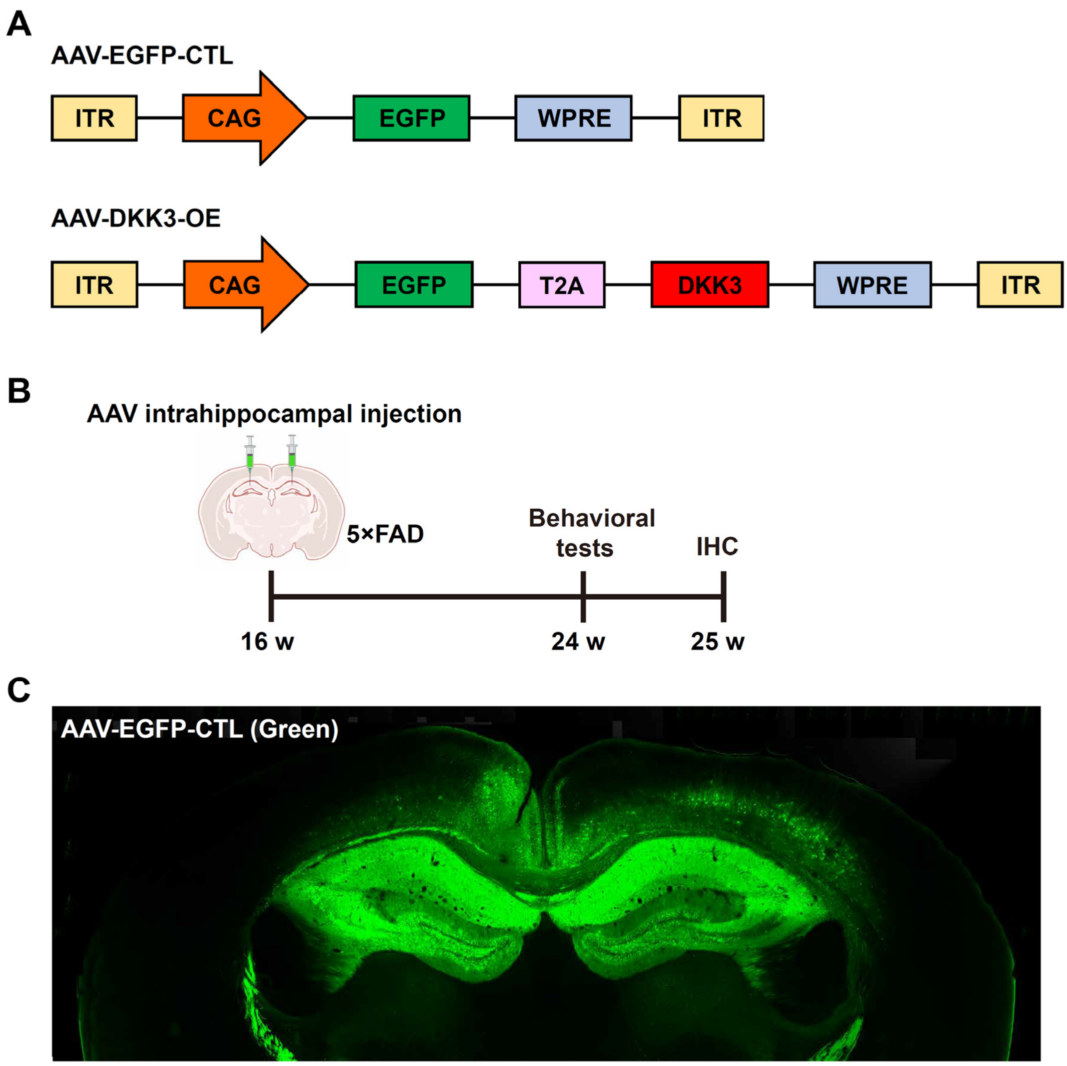

Supplementary Fig. S6 Adeno-associated viral (AAV)-mediated gene delivery

(**A**) Schematic structures of the adeno-associated viral (AAV) plasmids used in this study. (**B**) Experimental procedure of the AAV injection and subsequent animal studies. Created in BioRender. Yang, R. (2025) https://BioRender.com/y28wx4m. (**C**) Fluorescence image displaying enhanced green fluorescent protein (EGFP) expression in the mouse hippocampus, demonstrating the efficiency of AAV transduction.

Fig. S7.

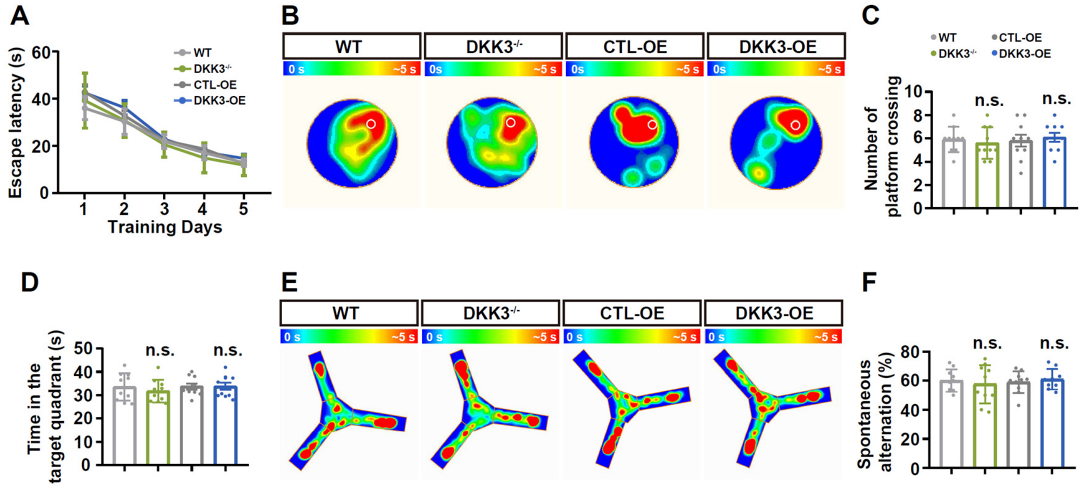

Supplementary Fig. S7 DKK3 knockout (-/-) or overexpression (OE) does not impair learning and memory in mice

(**A**) Graph displaying the escape latency for WT, DKK3$^{-/-}$, CTL-OE and DKK3-OE mice from Day 1 to Day 5 during Morris water maze (MWM) training. (**B**) Representative heatmap images from the probe trial in MWM tests at Day 6. Platform indicated as white circle. Quantitation shows the number of crossing the platform (**C**) and time spent (**D**) in the platform located quadrant. (**E**) Representative heatmap images showing the visit frequency of mice in Y-maze. (**F**) Quantification depicting the percentage of spontaneous alternation in (**E**). Data are presented as mean ± SD. Statistical significance was tested using Student's t-test, with n.s. denoting not significant.

Fig. S8.

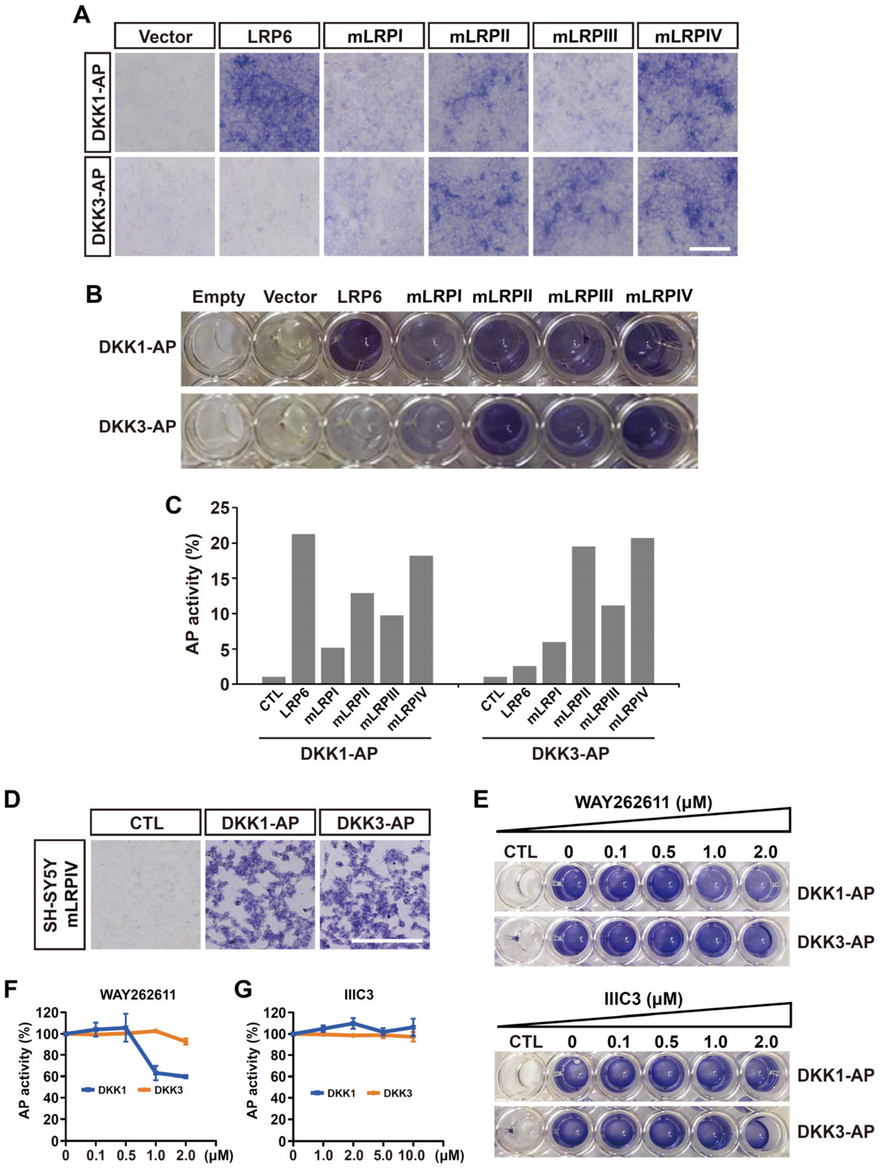

Supplementary Fig. S8 Cell surface binding assay shows measurable DKK-LRP binding affinity

(**A**) Cell surface AP-binding assays showing AP activity indicated by blue color intensity. 293 cells transfected with empty vector or vector expressing LRP6, or mLRPI-IV and then incubated with DKK1-AP CM or DKK3-AP CM for AP staining. Scale bar, 200 μm. (**B**) As in (**A**), but cells were lysed and AP stained. (**C**) Quantification of the enzymatic AP activity in (**B**) measured at A_{650}. The AP activity in empty vector transfected cells (CTL) normalized as 1.0. (**D**) AP staining

of mLRPIV$^+$ cells treated with CTL CM, DKK1-AP CM or DKK3-AP CM. Scale bar, 200 μm. (**E**) AP staining of cells as in (A), but following pre-incubation with indicated dosages of DKK inhibitors WAY262611 or IIIC3. (**F**, **G**) Quantified enzymatic AP activity in (E). The activity in cells treated with DMSO (control) was normalized to 100%.

Fig. S9.

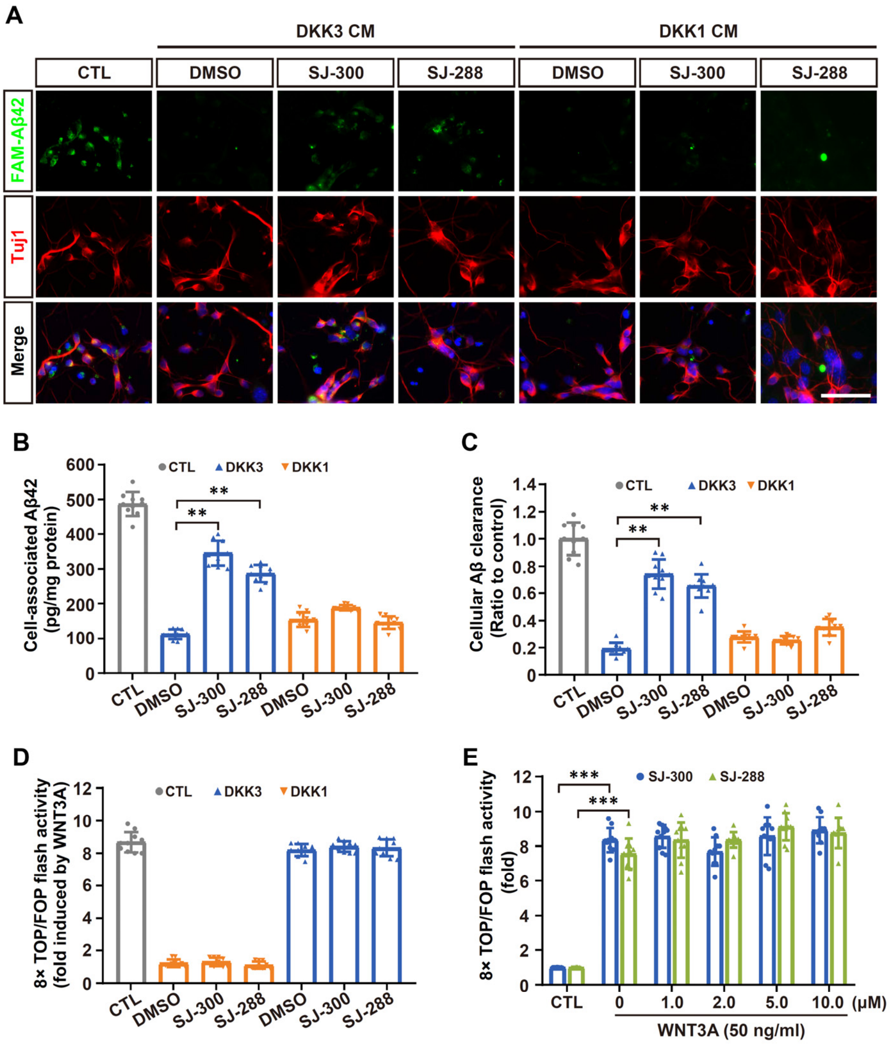

Supplementary Fig. S9 Both compounds reverse the DKK3 inhibition of Aβ clearance

(**A**) IF images showing differentiated AD-NSCs with indicated treatment as in Fig. 3D, but with the addition of 1 μM DMSO, SJ-300, or SJ-288 to either DKK3 or DKK1 CM. Green, internalized FAM-Aβ42; red, Tuj1-labeled neurons; blue, nuclei; Scale bar, 50 μm. (**B**) Quantification of cellular uptake of Aβ42 analyzed by ELISA as in Fig. 3E. (**C**) Cellular clearance of Aβ42 in each condition analyzed by ELISA as in Fig. 3F. (**D**) 8×TOP/FOP flash assays showing WNT/β-catenin activity. SH-SY5Y cells incubated with indicated CM supplemented with 10 μM DMSO, SJ-300 or SJ-288, followed by WNT3a (50 ng/mL) treatment. CTL CM treatment as baseline. Transfection and luciferase assays performed in biological triplicates. (**E**) As in (D), but varying

doses of SJ-300 or SJ-288 added without prior incubation with DKK1 or DKK3 CM. CTL, luciferase activity without WNT3a normalized as 1.0. Error bars, mean ± SD. $^{**}P<0.01$, $^{***}P < 0.001$; n ≥10 wells from at least 3 independent experiments; Student's *t*-test.

Fig. S10.

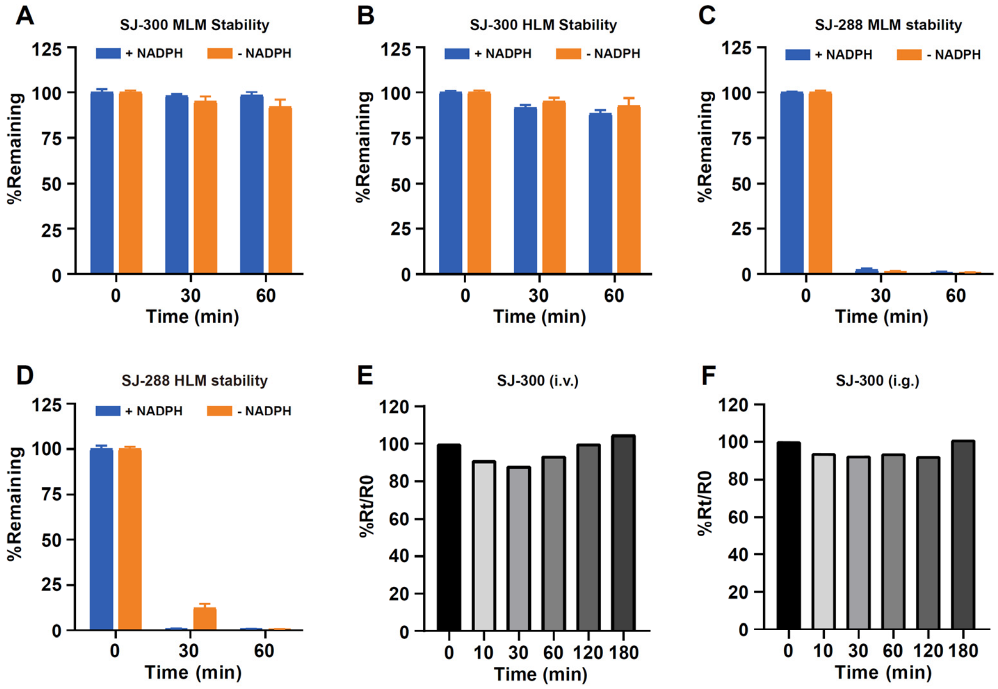

G

PK parameter	Unit	i.v. 2mg/kg	i.g. 10mg/kg
$t_{1/2}$	h	11.2	11.2
T_{max}	h	0.079	1
C_{max}	ng/mL	74.2	178
AUC_{0-24}	h*ng/mL	483	1721
AUC_{0-inf}	h*ng/mL	538	2280
Vz_F	mL/kg	52931	72973
Cl_F	mL/h/kg	3732	4687
MRT_{04}	h	7.2043	7.7965
F			71.30%

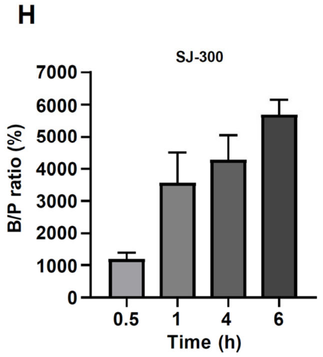

Supplementary Fig. S10 Pharmacokinetic analyses of SJ-300 and SJ-288 compounds

(**A, B**) Graphs depicting the metabolic stability of SJ-300 in mouse (A) and human (B) liver microsomes, both with and without the addition of NADPH. Quantitation showing the percentage of the remaining compound in liver microsomes over time. Data presented as mean ±SD (n=3 per time point). (**C**, **D**) Metabolic stability analysis of SJ-288. (**E**, **F**) Plasma concentration-time profiles of SJ-300 after i.v. (2 mg/kg, n = 6) or i.g. (10 mg/kg, n=6) administration. (**G**) Pharmacokinetic parameters of SJ-300 after i.v. (2 mg/kg) and i.g. (10 mg/kg). $t_{1/2}$, terminal half-life; C_{max}, maximum plasma concentration; T_{max}, time to reach C_{max}; $AUC_{0–24}$, area under the curve from 0–24 h; AUC_{0-inf}, area under the concentration–time curve from the time of dosing to infinity;

Vz_F, volume of distribution; Cl_F, clearance; MRT_{04}, mean residence time; F, oral bioavailability. (**H**) The ratio of brain/plasma concentration (B/P) time profiles of SJ-300. Error bars, mean ± SD. $n \geq 6$; Student's *t*-test.

Fig. S11.

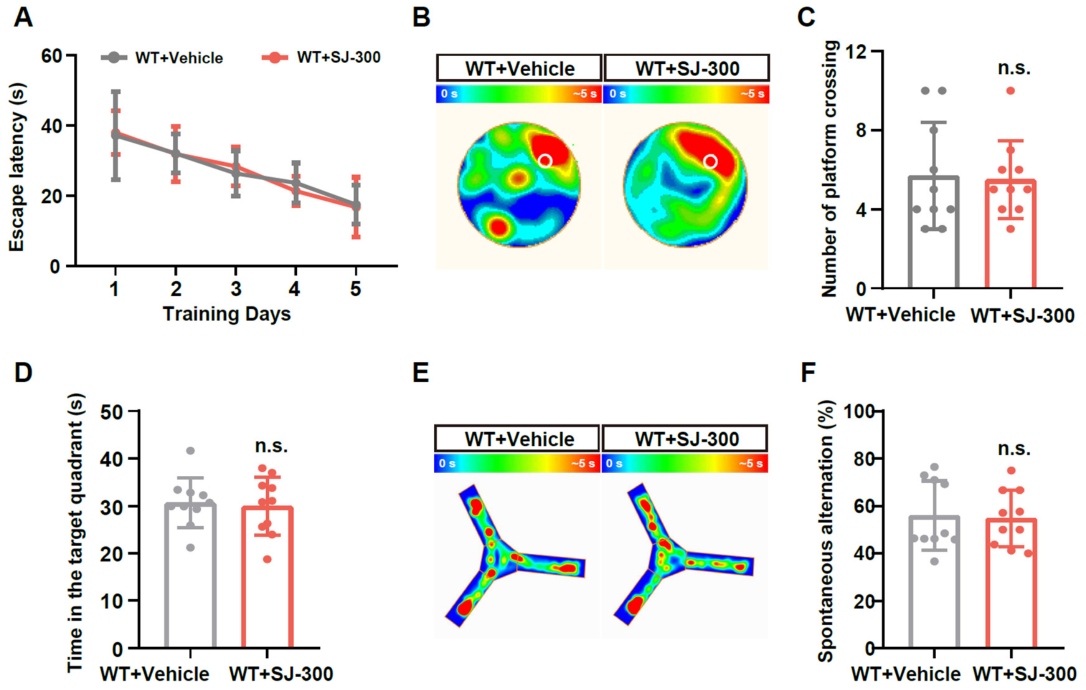

Supplementary Fig. S11 SJ-300 has no effect on the cognitive performance of WT mice

(**A**) Quantitation of the escape latency of mice from Day 1 to Day 5 during training. WT mice were randomly divided and intragastric (i.g.) administrated with vehicle or SJ-300 (5 mg/Kg) for 8 weeks (W) before behavioral tests. (**B**) Representative heatmap images of probe trials in MWM tests on Day 6. Platform indicated with white circle. Quantitation showing the number of crossing the platform (**C**) and time spent (**D**) in the platform located quadrant. (**E**) Representative heatmap images showing the visit frequency of mice in Y-maze. (**F**) Quantification showing the percentage of spontaneous alternation in (E). Error bars, mean ± SD. n=10 mice; n.s., not significant, Student's *t*-test.

Fig. S12.

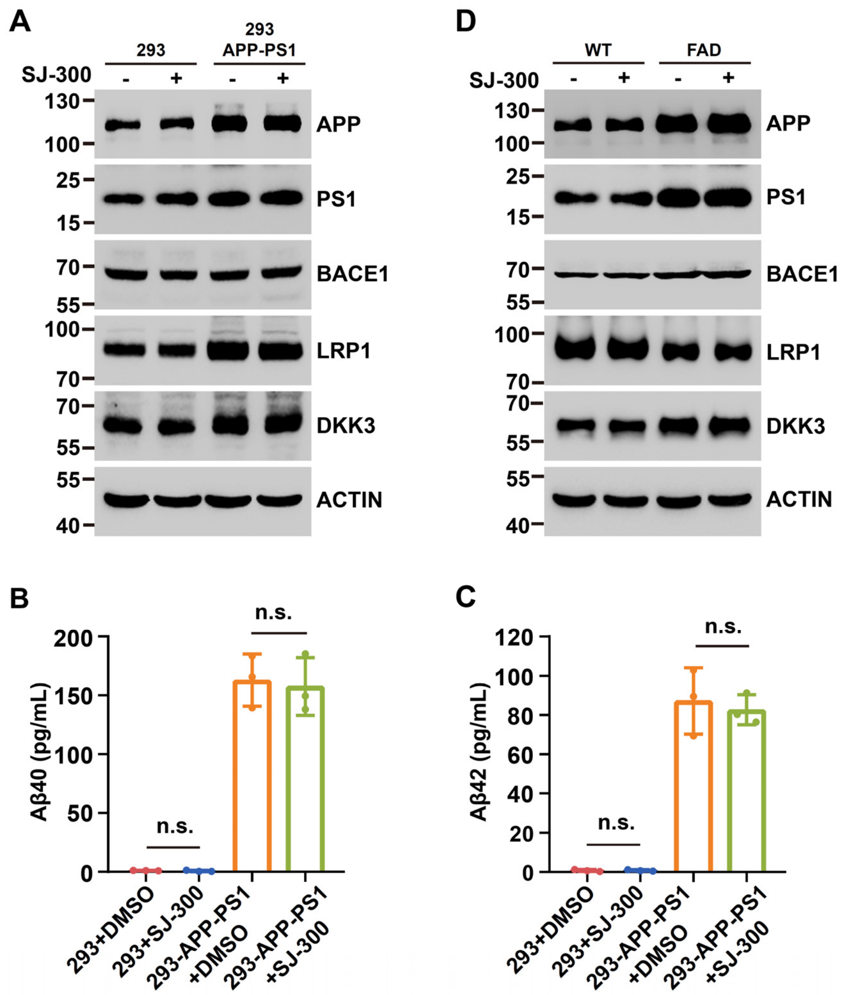

Supplementary Fig. S12 SJ-300 has no effect on APP cleavage and Aβ production

(**A**) Immunoblotting displaying levels of indicated proteins in naïve HEK293 cells and HEK293 cells stably expressing hAPP (with Swedish, Florida and London mutations) and hPS1 (with M146L and L286V mutations) after DMSO or SJ-300 (2 μM) treatment for 48 h. (**B, C**) ELISA showing the levels of Aβ40 (B) and Aβ42 (C) in culture media from indicated cell cultures and treatments. All data from at least 3 independent experiments. Error bars, mean ± SD; n.s., not significant, Student's *t*-test. (**D**) Immunoblotting displaying levels of indicated proteins from brains of 6 M WT and 5×FAD mice with vehicle or SJ-300 treatment for 8 W. Data from 3 independent experiments using protein lysate mixtures of each 5 mouse brain hemispheres.

Fig. S13.

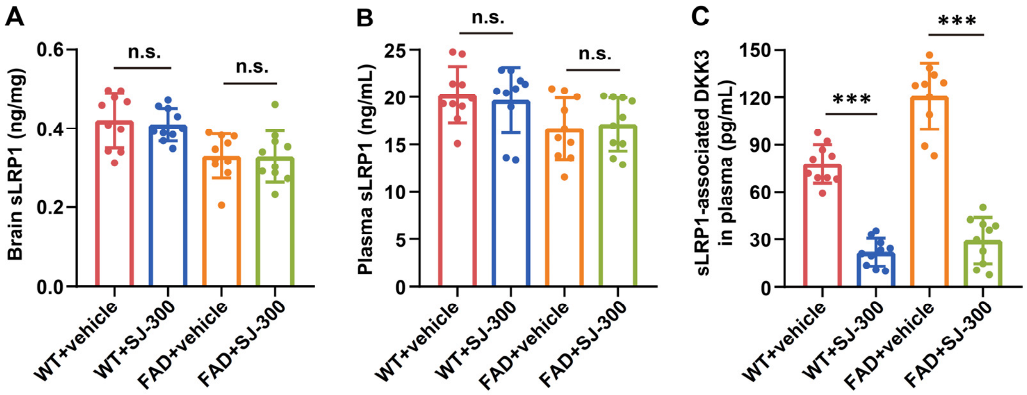

Supplementary Fig. S13 The effect of SJ-300 on levels of sLRP1 and sLRP1-associated DKK3

(**A, B**) ELISA showing levels of sLRP1 in brains (A) and in plasma (B) of WT and 5×FAD mice at 6 M old after 8 W treatment of vehicle or SJ-300. (**C**) As in (B), but focusing on the levels of sLRP1-associated DKK3 measured in plasma (see Methods). Error bars, mean ± SD. n=10 mice; n.s., not significant; ***$P < 0.001$; Student's *t*-test.

Table S1.

**Antibody**	**Vendor**
Anti-Actin (1:1000)	Bethyl Lab (#A300-491A)
Anti-Flag (1:1000)	Sigma-Aldrich (F1084)
Anti-HA (1:1000)	Cell signaling Tech (3724)
Anti-DKK4 (WB: 1:1000; IP: 1:200)	R&D Systems (AF3105)
Anti-LRP1 (WB: 1:5000; IP: 1:1000)	Abcam (ab92544)
Anti-LRP5 (1:1000)	Cell signaling Tech (5731)
Anti-LRP6 (1:1000)	Cell signaling Tech (3395)
Anti-DKK1 (WB: 1:1000; IP: 1:200)	Abcam (ab61034)
Anti-DKK2 (1:1000)	Cell signaling Tech (4689)
Anti-DKK3 (WB: 1:2000; WB: 1:500)	Abcam (ab186409)
Anti-Tju1 (1:2000)	Millipore (MAB1637)
Anti-GFAP (1:1000)	Abcam (ab7260)
Anti-NeuN (1:500)	Abcam (ab177487)
Anti-Iba1 (1:1000)	Wako (016-20001)
Anti-Aβ42 (1:1000)	Millipore (MABN12)
Anti-NaKATPase (1:1000)	Abcam (ab7671)
Anti-Flag affinity gel	Sigma-Aldrich (A2220)
Anti-HA affinity gel	Cell signaling Tech (3956)
Anti-CD31 (1:50)	BD Biosciences (550274)
Anti-6E10 (1:250)	Biolegend (803004)
Anti-APP (1:1000)	Proteintech (25524-1-AP)
Anti-PSEN1 (1:1000)	Cell signaling Tech (5743T)
Anti-BACE1 (1:1000)	Proteintech (12807-1-AP)
Anti-LRP1 (8G1) (1:500)	Abcam (ab20384)
Goat anti-Rabbit IgG (H+L) Alexa Fluor-568 (1:250)	Thermo (Cat#A11011)
Goat anti-Rabbit IgG (H+L) Alexa Fluor-633 (1:250)	Thermo (Cat#A21070)

Goat anti-Rat IgG (H+L) Alexa Fluor-546 (1:250)	Thermo (Cat#A11081)
Goat anti-Rabbit IgG (H+L) Alexa Fluor-488 (1:250)	Thermo (Cat#A11008)
Goat anti-Mouse IgG (H+L) Alexa Fluor-488 (1:250)	Thermo (Cat#A32723)
Donkey anti-Goat IgG (H+L) Alexa Fluor-488 (1:250)	Thermo (Cat#A-11055)
Goat Anti-Mouse IgG (H+L) HRP (1:250)	Thermo (Cat#31430)
Goat anti-Rabbit IgG (H+L) HRP (1:250)	Thermo (Cat#31460)
Rabbit anti goat IgG (H+L) HRP (1:250)	Proteintech (Cat#SA00001-4)
**Cell line**	**Vendor**
HEK293	ATCC (Cat#CRL-1573)
SH-SY5Y	ATCC (Cat#CRL-2266)
C8-D1A	ATCC (Cat#CRL-2541)
SIM-A9	ATCC (Cat#CRL-3265)
PEA-13	ATCC (Cat#CRL-2216)
Human iPSC-derived Neural Stem Cell (AD patient)	Axol Bioscience (Cat#ax0113)

Table S2.

Primer	Sequence
DKK4-F	AGCTAAGCTTGCCACCATGGTACTGGTGACCTTGCTTGGA
DKK4-R	GCGTGGATCCTATTCTTTGGCATACTCTTAGCC
DKK1-F	AGCTGGCCCAGCCGGCCATGATGGCTCTGGGCGCAGCGGGAGC
DKK1-R	GCGATCCGGAGTGTCTCTGACAAGTGTGAAGCCTA
DKK3-F	GCTAAGCTTACATGCAGCGGCTTGGGGCCACCCTGC
DKK3-R	GCGAAGATCTAATCTCTTCCCCTCCCAGCAGTGCA
hAPP-F	TCTGCTAACATGCGGTGACGTCGAGGAGAATCCTGGCCCAGCCACCATGCTGCCCGGTTTGGCACTGCTCC
hAPP-R	ATTAGGGCCCGTTCTGCATCTGCTCAAAGAACTTG
hPS1-F	ATTAACGCGTGCCACCATGACAGAGTTACCTGCACCGTTGT
hPS1-R	CCTCGACGTCACCGCATGTTAGCAGACTTCCTCTGCCCTCTTTGTCGTCATCATCCTTATAGTCC
mLRPIV-F	ATTAAAGCTTACCCCTGCAAGGTCAACAATGGTGGCT
mLRPIV-R	ATTACTCGAGCTTGTCGTCATCGTCTTTGTAGTCGGTCAGCTTGGGGTCGATGCTGCAG
human LRP1 sgRNA-F	CACCGATGCCAACGAGACCGTATGC
human LRP1 sgRNA-R	AAACGCATACGGTCTCGTTGGCATC

mouse LRP1 sgRNA-F	CACCGCGGCTCGGGACCCCACTGAGGGG
mouse LRP1 sgRNA-R	AAACCCCCTCAGTGGGGTCCCGAGCCGC
